# Liposomal Doxorubicin Induces PD-L1-High Tumor-Associated Macrophages and Sensitizes Triple-Negative Breast Cancer to PD-L1 Blockade

**DOI:** 10.32604/or.2026.087138

**Published:** 2026-09-14

**Authors:** Yu Zheng, Peng Zheng, Yidan Zheng, Zihan Xi, Tao Huang

**Affiliations:** 1 Department of Breast and Thyroid Surgery, Union Hospital, Tongji Medical College, Huazhong University of Science and Technology, Wuhan, China; 2 Department of Thyroid Surgery, Fujian Medical University Union Hospital, Fuzhou, China; 3 Department of Cardiovascular Surgery, Union Hospital, Tongji Medical College, Huazhong University of Science and Technology, Wuhan, China

**Keywords:** Liposomal doxorubicin, tumor-associated macrophages, programmed death-ligand 1 (PD-L1), triple-negative breast cancer, neoadjuvant chemotherapy

## Abstract

**Objective:** Liposomal doxorubicin (L-DOX) may alter macrophage-mediated immune regulation in triple-negative breast cancer (TNBC), but its role in programmed death-ligand 1 (PD-L1)-associated immune escape remains unclear. This study aimed to determine whether L-DOX induces a macrophage-centered PD-L1 response and affects the efficacy of PD-L1 blockade in TNBC. **Methods:** Public bulk and single-cell transcriptomic datasets, bone marrow-derived macrophage models, CD8^+^ T-cell co-culture assays, promoter-binding analyses, and syngeneic EO771 and 4T1 TNBC mouse models were used to examine PD-L1 regulation and immune function after L-DOX treatment. **Results:** The principal findings were that L-DOX preferentially induced a PD-L1-high macrophage state and sensitized TNBC tumors to PD-L1 blockade. DOX-containing therapy was associated with PD-L1 upregulation enriched in tumor-associated macrophages, and L-DOX induced stronger macrophage PD-L1 expression than free DOX or taxane-based agents. Mechanistically, L-DOX accumulation triggered DNA damage-associated ATM-p53 and cGAS-STING signaling, leading to RELA/p65-dependent NF-κB activation and PD-L1 transcription. Functionally, L-DOX-conditioned macrophages suppressed CD8^+^ T-cell activation, proliferation, and tumor-cell killing, whereas PD-L1 blockade restored CD8^+^ effector function, promoted pro-inflammatory macrophage features, and improved tumor control in EO771 and 4T1 models compared with either monotherapy. **Conclusion:** L-DOX induces a PD-L1-high macrophage state with immunosuppressive features that constrains antitumor T-cell responses. Combining L-DOX with PD-L1 blockade may provide a rational chemoimmunotherapy strategy for TNBC.

## 1 Introduction

Breast cancer remains a leading cause of cancer-related death among women worldwide [1]. Among its subtypes, triple-negative breast cancer (TNBC) lacks estrogen receptor, progesterone receptor, and HER2 expression and is associated with aggressive behavior, early metastasis, limited treatment options, and poor outcomes [2]. For locally advanced TNBC, neoadjuvant chemotherapy (NAC) is a standard treatment approach, aiming to reduce tumor burden, eradicate micrometastatic disease, and improve surgical outcomes [3].

Neoadjuvant chemotherapy has dual and potentially opposing immune effects on the tumor microenvironment (TME). Anthracyclines such as doxorubicin (DOX) induce immunogenic cell death to enhance antitumor immunity; however, they can also promote adaptive immune resistance by upregulating the expression of programmed death-ligand 1 (PD-L1) [4]. The upregulation of PD-L1 expression after chemotherapy is not limited to tumor cells but also occurs in immune cells, including macrophages, which may promote immune escape through the PD-1/PD-L1 axis [5,6]. Although anthracycline-based NAC regimens are widely used, the cell-specific regulatory mechanisms of PD-L1 during treatment and their functional consequences remain incompletely understood.

Liposomal doxorubicin (L-DOX), employing a polyethylene glycol-modified phospholipid bilayer, offers superior pharmacokinetics, enhanced tumor accumulation, and a significantly reduced risk of cardiotoxicity compared to conventional DOX [7]. Although doxorubicin has been reported to induce PD-L1 expression in tumor cells [8], liposomal formulation significantly alters the biodistribution of the drug: liposomal formulations are preferentially taken up by the mononuclear phagocyte system and accumulate in macrophages [9,10]. Tumor-associated macrophages (TAMs) are a major cellular source of PD-L1 in TME and play a central role in suppressing T cell function and conferring resistance to immunotherapy [11]. Whether the unique pharmacokinetics of L-DOX drive a macrophage-centric PD-L1 response, and which molecular mechanisms underlie this response, remain unclear.

Immune checkpoint blockade (ICB) therapies targeting the PD-1/PD-L1 axis have shown promise in TNBC, particularly when combined with chemotherapy [12]. However, it remains unclear whether L-DOX influences the therapeutic response to PD-L1 blockade by reshaping macrophage-mediated immunoregulation. Addressing this question is critical for optimizing chemoimmunotherapy strategies and identifying mechanistic biomarkers of response.

Therefore, this study aimed to determine whether L-DOX induces a macrophage-centered PD-L1 response in TNBC, to define the DNA damage-associated signaling mechanisms underlying this response, and to evaluate whether PD-L1 blockade can counteract L-DOX-associated macrophage-mediated immunosuppression.

## 2 Materials and Methods

### 2.1 RNA Sequencing Data Processing

To evaluate transcriptional changes associated with DOX-containing NAC or DOX treatment, publicly available bulk transcriptomic datasets were obtained from the Gene Expression Omnibus (GEO; https://www.ncbi.nlm.nih.gov/geo/) database. Human NAC-related datasets included GSE28583, GSE28826, GSE232764, and GSE260989, and murine DOX-treated breast cancer model datasets included GSE149479 and GSE133683. Among the human datasets, GSE28583, GSE28826, and GSE260989 contained matched pre- and post-chemotherapy breast tumor samples from the same patients, whereas GSE232764 contained paired baseline and post-run-in tumor biopsy samples. Detailed information for each dataset, including sample size, biological source, experimental groups, data type, and paired-sample design, is provided in Table S1.

All datasets were processed using a unified R-based pipeline (version 4.3.1; R Foundation for Statistical Computing, Vienna, Austria). For microarray datasets, GEO series matrix files were downloaded and probe IDs were mapped to official gene symbols using the corresponding platform annotation files. When multiple probes mapped to the same gene, the probe with the highest average expression was retained. For RNA-seq datasets, gene-level count or processed expression matrices were obtained from GEO and converted to gene symbols according to the provided annotation files. Genes without official gene symbols or genes detected in only one sample were removed. For each dataset, genes with low expression were filtered out by retaining genes with expression values greater than the lower quartile in at least 20% of samples. Expression values were then log_2_-transformed when necessary and normalized using the limma package (version 3.68.4). To harmonize datasets generated from different platforms, only common genes shared across datasets were retained for integration. Batch effects caused by dataset origin and platform differences were corrected using the ComBat function in the *sva* R package (version 3.58.0). The effectiveness of batch correction was assessed by principal component analysis (PCA) [13].

GSE116564 was analyzed to assess macrophage transcriptional changes following PD-L1 antibody treatment. This dataset contains RNA-seq profiles of murine bone marrow-derived macrophages treated for 24 h with an anti-PD-L1 antibody, an irrelevant isotype control antibody, or medium alone, with three biological replicates per group. The data were processed using the workflow described above.

### 2.2 Single-Cell RNA-Seq Data Processing

Single-cell RNA-seq (scRNA-seq) datasets were obtained from GEO, including two human TNBC datasets (GSE279219 and GSE266919) and one murine TNBC dataset (GSE191246). Detailed information regarding the sequencing platform, sample size, biological source, treatment conditions, and type of data obtained from GEO is provided in Table S2. GSE279219 comprised 24 TNBC tumor biopsy samples deposited in GEO, including 13 pretreatment samples and 11 samples collected after one cycle of doxorubicin plus cyclophosphamide-based neoadjuvant chemotherapy. GSE266919 comprised 78 biopsies from 44 TNBC patients treated with paclitaxel or nab-paclitaxel, with or without atezolizumab. GSE191246 comprised 18 murine tumor samples from the 4T1 and EMT6 models across untreated, chemotherapy, anti-PD-1, and combination-treatment conditions.

scRNA-seq data were processed using the Seurat package (version 5.5.1). Cells with fewer than 200 or more than 6000 detected genes, more than 40,000 unique molecular identifiers, greater than 20% mitochondrial transcripts, or greater than 5% hemoglobin transcripts were excluded. Gene-expression matrices were normalized using the LogNormalize method with a scale factor of 10,000, and the top 2000 highly variable genes were identified using the variance-stabilizing transformation method. The data were scaled and subjected to principal component analysis using the RunPCA function, with the first 30 principal components retained for downstream analysis.

Batch effects were corrected using the Harmony R package (version 2.0.5), with sample identity (orig.ident) specified as the integration variable and the PCA reduction used as input. Harmony was performed using dimensions 1–30, with theta = 2, lambda = 1, sigma = 0.1, and max.iter.harmony = 10. A shared nearest-neighbor graph was constructed from the Harmony-corrected dimensions 1–30 using the FindNeighbors function with k.param = 20. Graph-based clustering was performed using the Louvain algorithm implemented in the FindClusters function (algorithm = 1) at a resolution of 0.5. Cells were visualized by t-distributed stochastic neighbor embedding using the RunTSNE function based on Harmony dimensions 1–30, with a perplexity of 30. Cell types were annotated using canonical marker genes listed in Table S3.

### 2.3 Tumor Immune Infiltrate Analysis

Immune infiltration was quantified using both deconvolution-based and gene signature-based approaches [14]. The quanTIseq method was implemented using the immunedeconv R package (version 2.1.4) to estimate absolute immune cell fractions, and results were validated using CIBERSORT (version 1.03). The MCP-counter algorithm was applied using the MCPcounter R package (version 1.1.0) to infer relative changes in immune and stromal cell abundance based on the default transcriptomic marker gene sets. MCP-counter analysis was performed on the normalized and batch-corrected expression matrices generated by the unified preprocessing pipeline described above, and no additional quantile normalization was applied specifically for MCP-counter scoring. Duplicated genes within each signature were removed before scoring, and CD274 was excluded from the M2_Polarization signature to avoid circular interpretation when analyzing PD-L1-high macrophages. For marker heatmap analysis, RNA-assay data were LogNormalized in Seurat (version 5.5.1; scale factor = 10,000). Cluster-level mean expression was calculated using AverageExpression (assay = “RNA”, layer = “data”, and group.by = “seurat_clusters”) and visualized with pheatmap R package (version 1.0.13) after gene-wise z-score scaling across clusters (scale = “column”). Row and column clustering were disabled to preserve the predefined order. Representative markers were selected *a priori* from the published M1-like/pro-inflammatory and M2-like/immunoregulatory gene sets listed in Table S4. Duplicated genes were removed, and genes detected in at least 10% of cells in any macrophage cluster were retained. CD274 was displayed separately but excluded from phenotype scoring.

### 2.4 Cell Culture

Bone marrow cells were isolated from the femurs and tibias of 6–7-week-old mice, seeded at a density of 1 × 10^6^ cells/mL, and maintained in Dulbecco’s Modified Eagle Medium (DMEM) (Gibco, Thermo Fisher Scientific, 12491015, Waltham, MA, USA) containing 10% fetal bovine serum (FBS) (Gibco, Thermo Fisher Scientific, A5256701) and 10 ng/mL recombinant murine macrophage colony-stimulating factor (M-CSF) (MedChemExpress, HY-P7085, Monmouth Junction, NJ, USA), and were induced to differentiate into mature BMDMs over 7 days. Mature BMDMs were phenotypically characterized by flow cytometry as live CD11b^+^F4/80^+^ macrophages before subsequent experiments. Unless otherwise indicated, BMDMs were treated with 1 μM L-DOX for 24 h for mechanistic experiments. This condition was selected based on initial dose- and time-response optimization assays that assessed PD-L1 induction and BMDM viability. The *in vitro* concentration of anti-PD-L1 (Bio X Cell, BE0101, Lebanon, NH, USA) used for PD-L1 blockade was 100 μg/mL, which is the reported minimum effective plasma concentration [15]. Isotype control IgG (Bio X Cell, BE0090) was used to exclude nonspecific binding interference from antibodies. The inhibitors used in culture are listed in Table S5.

De-identified human peripheral blood monocytes (Procell Life Science & Technology Co., Ltd., CP-H182, Wuhan, Hubei, China) were commercially obtained and used to generate primary human monocyte-derived macrophages (hMDMs). No human participants were directly recruited, and no blood samples were collected by the authors. This work was conducted as part of a broader project approved by the Medical Ethics Committee of Union Hospital, Tongji Medical College, Huazhong University of Science and Technology ([2023] IEC (208)). Cells were cultured in human peripheral blood monocyte complete medium (Procell Life Science & Technology Co., Ltd., CM-H182) and differentiated into hMDMs with 10 ng/mL M-CSF (MedChemExpress, HY-P701101, Monmouth Junction, NJ, USA) for 7 days. Differentiated hMDMs were treated with DOX or L-DOX at 1 μM for 24 h, and PD-L1 expression was analyzed by flow cytometry in live CD45^+^CD11b^+^CD64^+^ cells. Detailed antibody information is provided in Table S6.

Bone marrow-derived dendritic cells (BMDCs) were generated by culturing bone marrow cells in Roswell Park Memorial Institute 1640 medium (RPMI 1640) (Gibco, Thermo Fisher Scientific, 11875093) supplemented with 10% FBS, 10 ng/mL granulocyte-macrophage colony-stimulating factor (PeproTech, 300-03, Rocky Hill, NJ, USA), and 10 ng/mL interleukin-4 (IL-4; PeproTech, 200-04) for 7 days.

CD8^+^ T cells were isolated from mouse spleens using a CD8^+^ T cell isolation kit (Elabscience, MIM003N, Wuhan, Hubei, China). Purified CD8^+^ T cells were resuspended at a density of 1 × 10^6^ cells/mL in medium consisting of RPMI 1640 with 10% FBS and 10 ng/mL recombinant murine interleukin-2 (IL-2; MedChemExpress, HY-P7077). T cells were activated using CD3/CD28 magnetic beads (Elabscience, MIM001A) and subsequently expanded until the culture volume increased approximately fourfold.

Murine breast cancer cell lines EO771 (ATCC, CRL-3461) and 4T1 (ATCC, CRL-2539) were obtained from the American Type Culture Collection (ATCC, Manassas, VA, USA) and maintained in DMEM with 10% FBS at 37°C in a humidified incubator containing 5% CO_2_. Both cell lines were authenticated by short tandem repeat (STR) profiling (Procell Life Science & Technology Co., Ltd., Wuhan, China), with matching scores of 96.29% and 97.86% for EO771 and 4T1, respectively, and were confirmed to be free of human cell cross-contamination. Cells were used within 10 passages after resuscitation.

Tumor-specific CD8^+^ T cells were generated as previously described [16]. Briefly, tumor cells were harvested and subjected to five freeze-thaw cycles (liquid nitrogen for freezing and a 37°C water bath for thawing). Cell debris was removed by centrifugation at 2000× *g* for 10 min at 4°C, and the resulting supernatant was collected and used to pulse BMDCs for 24 h. Antigen-pulsed BMDCs were then co-cultured with autologous CD8^+^ T cells and macrophages at a ratio of 1:5:5 for 5 days. Tumor-specific T cells were co-cultured with target tumor cells (EO771 or 4T1) at a 1:1 ratio for 12 h to assess tumor cell killing capacity. L-DOX was used at 1 μM for macrophage conditioning, and anti-PD-L1 or matched IgG control was used at 100 μg/mL during co-culture.

The chemotherapeutic agents used in this study included liposomal doxorubicin (L-DOX), doxorubicin (DOX), paclitaxel (PTX), docetaxel (DTX), nab-paclitaxel (Nab-PTX), and liposomal paclitaxel (L-PTX). Detailed information on sources, catalog numbers, and working concentrations is provided in Table S5. All cultured cells were tested for mycoplasma contamination using the LookOut^®^ Mycoplasma PCR Detection Kit (Sigma-Aldrich, MP0035, St. Louis, MO, USA) according to the manufacturer’s instructions and were confirmed to be mycoplasma-free before use.

### 2.5 Western Blotting

Western blotting was performed as previously described [16]. Total proteins were extracted using RIPA Lysis Buffer (Beyotime Biotechnology, P0013K, Shanghai, China) supplemented with Protease Inhibitor Cocktail for General Use (100×) (Beyotime Biotechnology, P1005) and Phosphatase Inhibitor Cocktail A (50×) (Beyotime Biotechnology, P1081). Protein concentrations were determined using an Enhanced BCA Protein Assay Kit (Beyotime Biotechnology, P0010). For all target proteins, 30 μg of total protein per lane was separated by 10% SDS-PAGE and transferred to PVDF membranes (Beyotime Biotechnology, FFP70). Membranes were incubated with the primary antibodies listed in Table S7 overnight at 4°C. After three 10-min washes with TBST, membranes were incubated with HRP-conjugated anti-rabbit or anti-mouse IgG secondary antibodies (Cell Signaling Technology, 7074 and 7076, Danvers, MA, USA; 1:2000) for 1 h at room temperature. Signals were developed using an enhanced chemiluminescence substrate (Thermo Fisher Scientific, 32106) for 5 min and captured using a ChemiDoc XRS+ Imaging System (Bio-Rad Laboratories, Inc., 1708265, Hercules, CA, USA). Band intensities were quantified after background subtraction using ImageJ software (version 1.54p; National Institutes of Health, Bethesda, MD, USA). Phosphorylated proteins were normalized to their corresponding total proteins, whereas other target proteins were normalized to β-actin.

### 2.6 Flow Cytometry

For surface staining, single-cell suspensions were stained with a fixable viability dye and blocked with anti-CD16/CD32, followed by antibody incubation. For intracellular detection of CD206, IFN-γ, and GZMB, surface-stained cells were fixed and permeabilized using Cytofix/Cytoperm solution (BD Biosciences, 554714, Franklin Lakes, NJ, USA) for 20 min at 4°C, washed twice with kit-provided Perm/Wash buffer, and incubated with the corresponding intracellular antibodies for 30 min at 4°C in the dark. For proliferation analysis, purified CD8^+^ T cells were resuspended at 1 × 10^6^ cells/mL and labeled with 5 μM carboxyfluorescein succinimidyl ester (CFSE; Elabscience, E-CK-A345) for 10 min at 37°C in the dark. Labeling was terminated by adding five volumes of complete culture medium, followed by two washes with complete medium. CFSE-labeled CD8^+^ T cells were co-cultured with control- or L-DOX-conditioned macrophages at a macrophage-to-T-cell ratio of 1:1 for 72 h, and proliferation was assessed at 72 h based on CFSE dilution. Data were acquired on a FACSVerse flow cytometer (BD Biosciences, Franklin Lakes, NJ, USA) and analyzed with FlowJo software (version 10.8.1; BD Life Sciences, Ashland, OR, USA). Compensation was performed using single-stained controls acquired under identical instrument settings. Debris, doublets, and dead cells were excluded sequentially, and positive gates were defined using fluorescence-minus-one controls. At least 10,000 live singlet events were analyzed per sample, with results reported as population frequencies, mean fluorescence intensity, or CFSE-diluted cell percentages, as appropriate. Data represent at least three independent experiments; antibody details are provided in Table S6.

### 2.7 Immunofluorescence Staining

BMDMs were seeded on glass coverslips in 24-well plates at 5 × 10^4^ cells per well. After the indicated treatments, cells were washed with PBS, fixed with 4% paraformaldehyde (Servicebio, G1101, Wuhan, Hubei, China) for 30 min at room temperature, permeabilized with 0.1% Triton X-100 (Sigma-Aldrich, T8787, St. Louis, MO, USA) for 10 min, and blocked with 5% bovine serum albumin (Servicebio, G5001) for 1 h. Cells were incubated with anti-γ-H2A.X primary antibody (Cell Signaling Technology, 80312S, Danvers, MA, USA; 1:200) overnight at 4°C, followed by the corresponding Alexa Fluor 488-conjugated goat anti-mouse IgG secondary antibody (Thermo Fisher Scientific, A-11001; 1:500) for 1 h at room temperature in the dark. Secondary antibody-only controls were included. Nuclei were stained using DAPI-containing mounting medium (Cell Signaling Technology, 4083S). Images were acquired using a Nikon A1 laser-scanning confocal microscope (Nikon Corporation, Nikon A1, Tokyo, Japan) equipped with a 60× oil-immersion objective, using 405- and 488-nm excitation and a resolution of 1024 × 1024 pixels. Identical acquisition settings were applied to all groups. Images were acquired and processed using NIS-Elements AR software (version 5.01; Nikon Corporation).

### 2.8 Assessment of Drug Uptake Capacity

Cellular drug uptake was estimated by measuring the reduction in residual drug fluorescence in the culture supernatant. Mature BMDMs were seeded in 96-well plates at 1 × 10^4^ cells per well and allowed to adhere overnight. Cells were then treated with L-DOX, DOX, PTX, DTX, Nab-PTX, or L-PTX at 0.1, 0.5, 1, or 2 μM for 12 h at 37°C in 5% CO_2_. Drug-free medium and supernatants from untreated cells were used as background controls. For each agent and concentration, cell-free wells containing the corresponding drug were incubated in parallel under identical conditions to account for drug degradation, formulation-dependent fluorescence, and concentration-dependent fluorescence quenching.

Doxorubicin fluorescence was measured at excitation/emission wavelengths of 470/560 nm using a Synergy H1 microplate reader (BioTek Instruments, Synergy H1, Winooski, VT, USA). Fluorescence of PTX, DTX, Nab-PTX, and L-PTX was measured at excitation/emission wavelengths of 230 nm/340 nm using a Cary Eclipse fluorescence spectrophotometer equipped with a microplate reader accessory (Agilent Technologies, Cary Eclipse, Santa Clara, CA, USA) [17,18]. The relative uptake ratio was calculated as follows:

Relative uptake ratio (%) = [1 − (Fsample − Fcell blank)/(Fcell-free drug − Fmedium blank)] × 100

where Fsample represents the fluorescence of supernatants from drug-treated BMDMs, Fcell blank represents the fluorescence of supernatants from untreated BMDMs, Fcell-free drug represents the fluorescence of the corresponding drug incubated without cells, and Fmedium blank represents the fluorescence of drug-free medium. Each drug and concentration was normalized to its corresponding cell-free control. Data were obtained from five independent experiments.

### 2.9 Cell Viability Assay

BMDMs were seeded into 96-well plates at 1 × 10^4^ cells per well and allowed to adhere overnight. For the dose-response assay, cells were treated with L-DOX at 0, 0.1, 0.5, 1, or 2 μM for 24 h. For the time-response assay, cells were treated with 1 μM L-DOX for 0, 12, 24, 36, or 48 h. Subsequently, 10 μL of Cell Counting Kit-8 (Solarbio, CA1210, Beijing, China) was added to each well containing 100 μL of culture medium and incubated for 2 h at 37°C. Blank wells containing culture medium and CCK-8 reagent without cells were included for background correction. Absorbance was measured at 450 nm using a Synergy H1 microplate reader (BioTek Instruments, Winooski, VT, USA). Relative cell viability was calculated as follows:

Relative cell viability (%) = [(A_treated − A_blank)/(A_control − A_blank)] × 100,

where A_treated, A_control, and A_blank represent the absorbance values of L-DOX-treated cells, untreated control cells, and blank wells, respectively. The 0 μM group and the 0 h group were used as the normalization references for the dose- and time-response assays, respectively. Data are presented as pooled results from three independent biological experiments, each performed with three technical replicate wells.

### 2.10 Transcriptome Sequencing and Analysis

For *in vitro* transcriptome sequencing, BMDMs were treated with L-DOX (1 μM, 24 h) or vehicle control, with four biologically independent samples per group. Total RNA was extracted using TRIzol reagent (Invitrogen, Thermo Fisher Scientific, 15596026CN) and subjected to transcriptome RNA-seq as previously described [19]. Poly(A)-enriched, strand-specific RNA-seq libraries were prepared by Majorbio Bio-Pharm Technology Co., Ltd. (Shanghai, China) and sequenced on an Illumina NovaSeq 6000 sequencing system (Illumina, Inc., San Diego, CA, USA) using 150-bp paired-end reads, generating approximately 40 million clean reads per sample. The generated RNA-seq data have been deposited in the China National Center for Bioinformation under accession number PRJCA052710.

Raw gene-level read counts were analyzed using the edgeR package (version 4.0.16) from Bioconductor (version 3.18) running on R (version 4.3.0). Low-expression genes were removed using the filterByExpr function, and library sizes were normalized using the trimmed mean of M-values method. Differential expression was assessed using a negative-binomial generalized linear model and quasi-likelihood F-test. Differentially expressed genes were defined as those with an absolute log2 fold change greater than 1 and a Benjamini–Hochberg-adjusted false discovery rate below 0.05.

Functional enrichment analyses, including Gene Ontology (GO), Kyoto Encyclopedia of Genes and Genomes (KEGG) pathway analysis, and Gene Set Enrichment Analysis (GSEA), were conducted using the clusterProfiler package (version 4.10.0) with gene sets obtained from MSigDB version 2023.2.Mm, including the senescence-associated secretory phenotype (SASP) gene set [20]. GSEA was performed with 1000 permutations, an exponent of 1, minimum and maximum gene-set sizes of 10 and 500, respectively, and Benjamini–Hochberg-adjusted FDR < 0.05. Transcription factor target enrichment was assessed using MSigDB C3 (TFT) gene sets and a hypergeometric test, with all expressed genes used as the background. *p* values were adjusted using the Benjamini–Hochberg method, and gene sets with FDR < 0.05 were considered significantly enriched. For significantly enriched TFs, Pearson correlations between log2-CPM expression of the TF and *Cd274* were calculated across all eight BMDM RNA-seq samples, including four control and four L-DOX-treated samples; two-sided *p* < 0.05 was considered statistically significant.

### 2.11 Senescence β-Galactosidase Staining

BMDMs were treated with L-DOX (1 μM) or vehicle control for 24 h and stained using a Senescence β-Galactosidase Staining Kit (Beyotime Biotechnology, C0602, Shanghai, China). Cells were washed twice with PBS, fixed with the kit-provided fixation solution for 15 min at room temperature, washed three times with PBS, and incubated with freshly prepared staining solution overnight at 37°C in a non-CO_2_ incubator. Cells exhibiting distinct blue cytoplasmic staining were considered β-galactosidase-positive. Images were acquired using a Nikon Eclipse Ts2 inverted microscope (Nikon Corporation, Eclipse Ts2, Tokyo, Japan) with a 20× objective. Positive and total cells were manually counted using the Cell Counter plugin in ImageJ software (version 1.54p; National Institutes of Health, Bethesda, MD, USA) in five randomly selected fields per sample. The percentage of β-galactosidase-positive cells was calculated as the number of positive cells divided by the total number of cells × 100%. Data were pooled from three independent experiments.

### 2.12 Dual-Luciferase Reporter Assay

Dual-luciferase reporter assays were performed using the Dual-Luciferase Reporter Assay Kit (Beyotime Biotechnology, RG027). Reporter constructs contained a 2100-bp fragment of the mouse *Cd274* promoter spanning −2000 to +100 bp relative to the transcription start site. Three JASPAR-predicted NF-κB-binding sites were individually mutated: p1 at −1980 to −1971 bp (AGGAAATCCC to ATTAGGACCC), p2 at −1164 to −1155 bp (GGGAAATCTC to CCTTTTAGAG), and p3 at −475 to −466 bp (GGTATTTCCC to TTTCGGACCC). The wild-type and mutant promoter fragments were synthesized and cloned into the pGL3-basic vector by Tsingke Biotechnology Co., Ltd. (Beijing, China). The pRL-TK Renilla luciferase control plasmid (Promega Corporation, E2241, Madison, WI, USA) was used as an internal control. BMDMs cultured in 24-well plates were co-transfected with 500 ng of reporter plasmid and 50 ng of pRL-TK per well using 1.5 μL of Lipofectamine 3000 (Thermo Fisher Scientific, L3000001). After 24 h of transfection, cells were subjected to the indicated treatments for an additional 24 h. Cells were then lysed, and Firefly and Renilla luciferase activities were sequentially measured using a Synergy H1 multimode microplate reader (BioTek Instruments) in luminescence mode according to the kit instructions. Firefly luciferase activity was normalized to Renilla luciferase activity. Each condition was examined in three independent biological experiments, each performed in three technical replicate wells, and technical replicates were averaged before statistical analysis.

### 2.13 Chromatin Immunoprecipitation (ChIP)-qPCR

ChIP assays were performed using the EZ-Magna ChIP A/G Kit (MilliporeSigma, 17-10086, Burlington, MA, USA) as previously described [21]. BMDMs (1 × 10^7^ cells per condition) treated with L-DOX (1 μM) or vehicle control for 24 h were crosslinked with 1% formaldehyde for 10 min at room temperature, followed by quenching with 125 mM glycine for 5 min. Chromatin was sonicated on ice using six 15-s pulses separated by 50-s cooling intervals, yielding DNA fragments predominantly between 200 and 1000 bp.

Chromatin corresponding to approximately 1 × 10^6^ cells was used for each immunoprecipitation, and 1% of each chromatin preparation was retained as the input control. Immunoprecipitation was performed overnight at 4°C using 5 μg of anti-p65 antibody (Thermo Fisher Scientific, 51-0500) or 5 μg of normal rabbit IgG (Thermo Fisher Scientific, 02-6102). The anti-p65 antibody has been validated by the manufacturer for ChIP applications, with 5 μg recommended for ChIP assays.

After washing, elution, reverse crosslinking, and DNA purification, enriched DNA was quantified using ChamQ Universal SYBR qPCR Master Mix (Vazyme Biotech, Q711, Nanjing, Jiangsu, China) on a QuantStudio 5 Real-Time PCR System (Applied Biosystems, Thermo Fisher Scientific, QuantStudio 5, Waltham, MA, USA). Primer pairs encompassing the three JASPAR-predicted NF-κB-binding sites within the mouse *Cd274* promoter were used, and their sequences, genomic regions, and amplicon sizes are provided in Table S8. ChIP-qPCR primers were synthesized by Sangon Biotech (Shanghai) Co., Ltd., Wuhan Branch (Wuhan, Hubei, China).

ChIP enrichment was calculated using the percentage-of-input method and presented as fold enrichment relative to the corresponding IgG control. Three independent biological experiments were performed, with each qPCR reaction analyzed in three technical replicates. Technical replicates were averaged before statistical analysis.

### 2.14 Electroporation and siRNA-Mediated Knockdown

BMDMs were transfected with p65-targeting siRNAs or negative control siRNA synthesized by GenePharma Co., Ltd. (Shanghai, China) using a Nucleofector 2b Device (Lonza, Nucleofector 2b, Basel, Switzerland) according to a previously established protocol [16]. For each electroporation, 1 × 10^6^ BMDMs were resuspended in 100 μL of supplemented Mouse Macrophage Nucleofector Solution (82 μL Nucleofector Solution and 18 μL Supplement; Lonza, VPA-1009) containing 100 nM siRNA. Electroporation was performed using program Y-001. Cells were immediately transferred to prewarmed complete medium. At 48 h post-electroporation, knockdown efficiency was assessed by western blotting, and cells were used for downstream functional assays. Knockdown efficiency was confirmed by western blotting, and the siRNA sequences are listed in Table S9.

### 2.15 In Vivo Experiments

Mice were randomly assigned to treatment groups using a computer-generated random number sequence. A total of 40 six-week-old female mice, including 20 C57BL/6 and 20 BALB/c mice, weighing 18–22 g, were provided by the Experimental Animal Center of Huazhong University of Science and Technology (Wuhan, Hubei, China) and maintained under specific pathogen-free conditions. All mice were wild-type animals with no prior experimental procedures and were acclimatized for at least 1 week before tumor inoculation. Mice were housed under a 12-h light/dark cycle with free access to standard chow and water. EO771 cells in C57BL/6 mice and 4T1 cells in BALB/c mice were inoculated into the mammary fat pads at 1 × 10^5^ cells in 50 μL of sterile PBS per mouse under isoflurane inhalation anesthesia (RWD Life Science Co., Ltd., R510-22-8, Shenzhen, Guangdong, China), following institutional guidelines. Treatments began when tumors reached ~100 mm^3^. For each tumor model, mice were allocated to four treatment groups, with five mice per group. Each mouse bearing one tumor was considered one experimental unit.

Mice received L-DOX (Changzhou Jinyuan Pharmaceutical Manufacturing Co., Ltd., NMPA approval No. H20123273, Changzhou, Jiangsu, China) at 5 mg/kg intraperitoneally once weekly. Anti-PD-L1 antibody (Bio X Cell, BE0101) was administered intraperitoneally twice weekly at 10 mg/kg for the first dose and 5 mg/kg for subsequent doses. The matched isotype-control IgG (Bio X Cell, BE0090) was administered on the same schedule and at the corresponding doses. All intraperitoneal treatments were administered in a total injection volume of 100 μL per mouse. All treatment groups were handled and treated in parallel to minimize potential confounding from treatment order. Mice were housed in the institutional SPF barrier facility under centrally controlled environmental conditions. Cages were not rotated or randomly repositioned during the experiment, and cage positions were not deliberately assigned according to treatment group. Tumor size was measured every three days using calipers, and tumor volume was calculated as 1/2 × length × width^2^. Tumor volume was defined as the primary outcome. Humane endpoints included tumor volume approaching 1500 mm^3^, any single tumor dimension exceeding 15 mm, tumor ulceration, impaired mobility, severe distress, or body-weight loss exceeding 20%. Experiments were terminated when the maximum tumor volume in the control group reached approximately 1500 mm^3^, with no single tumor dimension exceeding 15 mm. At the experimental endpoint, mice were euthanized in a gradually filled CO_2_ chamber at a displacement rate of approximately 30% of the chamber volume per minute. CO_2_ flow was maintained for at least 1 min after respiratory arrest. Death was confirmed by the absence of respiration and heartbeat, followed by cervical dislocation as a secondary method, before tissue collection. No animals were excluded from allocation, treatment, or final analysis.

Tumors were harvested for weight measurement and immune profiling by flow cytometry. Tumors were digested in RPMI 1640 containing collagenase IV (Servicebio, GC305015-100 mg) and DNase I (Servicebio, G3342-500U). Cell suspensions were passed through 70-μm cell strainers (Biosharp, BS-70-CS, Beijing, China) and treated with red blood cell lysis buffer (Servicebio, G2015-500 ML). Live singlet CD45^+^ cells were selected for immune-cell analysis. Tumor-associated macrophages were identified as CD45^+^CD11b^+^F4/80^+^ cells and analyzed for PD-L1, CD86, and CD206 expression. CD8^+^ T cells were identified as CD45^+^CD3^+^CD8^+^ cells and analyzed for CD69, PD-1, TIM-3, IFN-γ, and GZMB expression. Tumor cells were identified as live CD45^−^EpCAM^+^ cells. Compensation was performed using single-stained controls, and positive gates were defined using fluorescence-minus-one controls. Data were analyzed using FlowJo software (version 10.8.1). Antibody information is provided in Table S6.

Tumor measurements and flow cytometry analyses were performed by investigators blinded to treatment allocation. All procedures complied with institutional animal care guidelines. All animal experiments were approved by the Institutional Animal Care and Use Committee of Huazhong University of Science and Technology (HUST-IACUC-2025-0016) and were conducted in accordance with institutional guidelines and the ARRIVE 2.0 guidelines.

### 2.16 Statistical Methods

Data are presented as mean ± SD unless otherwise indicated. The number of biologically independent replicates, animals, or patient samples is indicated in the figure legends. No technical replicates were treated as independent biological replicates. For comparisons between two groups, two-tailed paired or unpaired Student’s *t*-test was used as appropriate. Welch’s *t*-test was used when unequal variance or unequal group distribution was expected. For comparisons among more than two groups, one-way ANOVA followed by Tukey’s, Dunnett’s, or Sidak’s multiple-comparison correction was used as indicated in the figure legends. Tumor growth curves were analyzed by two-way repeated-measures ANOVA with Sidak’s multiple-comparison correction. Correlations were assessed using Pearson or Spearman correlation according to data distribution. Hypergeometric tests were used for transcription factor enrichment analysis. For transcriptomic analyses, adjusted *p* values or false discovery rate (FDR) values were used where applicable. *p* < 0.05 was considered statistically significant.

## 3 Results

### 3.1 DOX-Containing NAC Is Associated with PD-L1 Upregulation in TAMs

**Figure 1:**
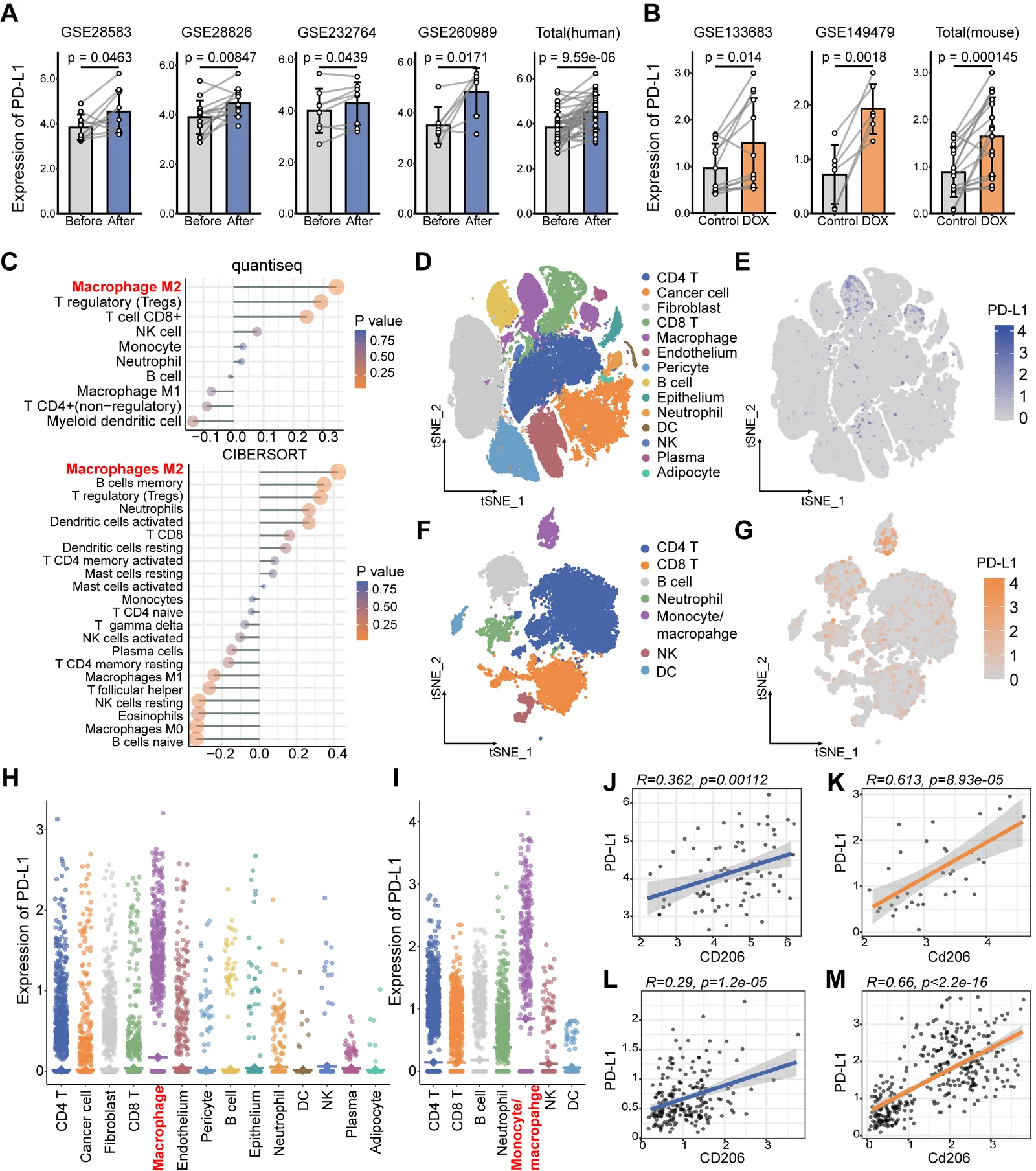
**DOX-containing NAC upregulates PD-L1 expression in macrophages.** (**A**,**B**) PD-L1 expression levels in bulk RNA-seq datasets from breast cancer patients (**A**) and DOX-treated murine breast cancer models (**B**) after normalization and batch effect correction. (**C**) Correlation between the abundance of various immune cell types (deconvoluted using quanTIseq/CIBERSORT algorithms) and PD-L1 expression in bulk RNA-seq data from breast cancer patients. (**D**) t-SNE visualization and (**E**) PD-L1 expression feature plot of GSE279219. (**F**) t-SNE visualization and (**G**) PD-L1 expression feature plot of GSE191246. (**H**,**I**) PD-L1 expression levels across different immune cell subsets in the GSE279219 (**H**) and GSE191246 (**I**) datasets (diamonds and horizontal lines represent mean values). (**J**,**K**) Scatter plots showing the correlation between PD-L1 and CD206 expression in human (**J**) and murine (**K**) bulk RNA-seq data. (**L**,**M**) Scatter plots showing the correlation between PD-L1 and CD206 expression in cells with non-zero gene expression from human (**L**) and murine (**M**) scRNA-seq data. Statistical tests: two-tailed paired Student’s *t*-test (**A**); two-tailed unpaired Student’s *t*-test (**B**); Spearman correlation test (**C**); Pearson correlation test with linear regression fit (**J**–**M**). Abbreviations: NAC, neoadjuvant chemotherapy; DOX, doxorubicin; PD-L1, programmed death-ligand 1; RNA-seq, RNA sequencing; scRNA-seq, single-cell RNA sequencing; GSE, gene expression omnibus series; t-SNE, t-distributed stochastic neighbor embedding; quanTIseq, quantification of the tumor immune contexture from RNA-seq data; CIBERSORT, cell-type identification by estimating relative subsets of RNA transcripts; Treg, regulatory T cell; NK, natural killer; DC, dendritic cell; M0, unpolarized macrophage phenotype; M1, M1-like/pro-inflammatory macrophage phenotype; M2, M2-like/immunoregulatory macrophage phenotype.

We integrated paired bulk transcriptomic datasets from GEO to evaluate transcriptional changes induced by DOX-containing NAC. After normalization and batch-effect correction (Fig. S1A), PD-L1 expression was significantly upregulated after DOX treatment in both human breast cancer samples and murine models (Fig. 1A,B).

To identify the cellular source of PD-L1 induction, we deconvoluted the tumor immune microenvironment using two independent algorithms, quanTIseq and CIBERSORT (Fig. S1B). Both approaches revealed a significant positive correlation between PD-L1 expression and M2-like macrophage abundance (Fig. 1C and Fig. S1C). In addition, MCP-counter analysis indicated that DOX-containing NAC was associated with increased macrophage infiltration in both human and murine datasets (Fig. S1D,E).

To further resolve PD-L1 expression at single-cell resolution, we analyzed scRNA-seq datasets from DOX-treated TNBC patients (GSE279219) and murine models (GSE191246). Cross-species single-cell analyses revealed DOX-induced upregulation of human *CD274* and murine *Cd274* in monocyte-macrophage clusters (Fig. 1D–G and Fig. S1F–I). Quantitative comparisons across immune cell subsets confirmed that TAMs exhibited markedly higher PD-L1 expression than other immune populations in both human and murine datasets (Fig. 1H,I).

Given the role of PD-L1 in immune suppression, we next examined whether PD-L1-high macrophages displayed additional immunoregulatory features. Both bulk and single-cell analyses revealed positive correlations between *CD274* and *MRC1* expression in human samples and between *Cd274* and *Mrc1* expression in murine samples (Fig. 1J–M). These analyses suggest that DOX-containing NAC is associated with preferential PD-L1 upregulation in TAMs with immunosuppressive features.

### 3.2 L-DOX Potently Induces PD-L1 Upregulation in Macrophages

**Figure 2:**
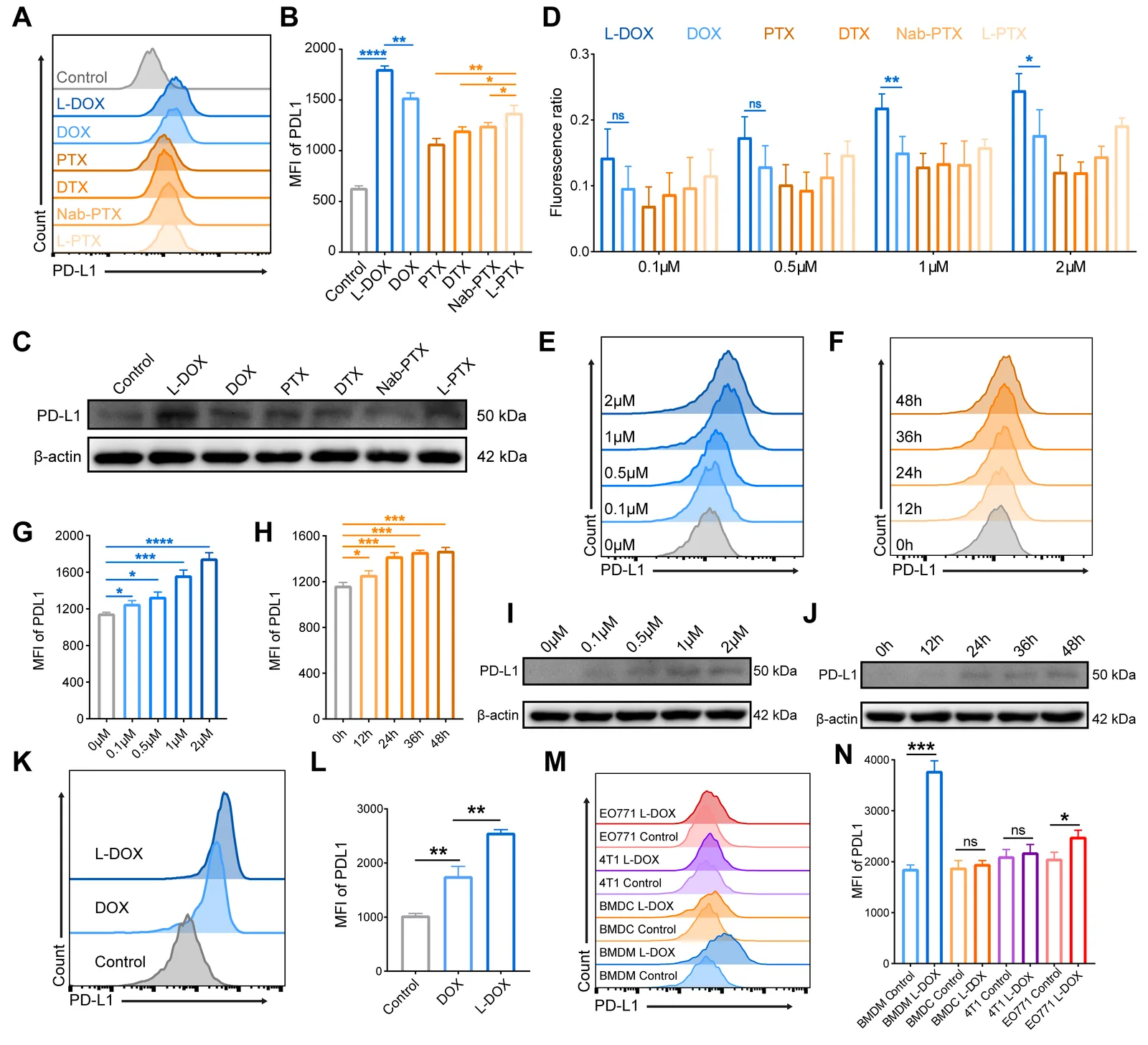
**L-DOX potently induces PD-L1 upregulation in macrophages.** (**A**) Representative flow cytometry histograms showing PD-L1 expression on BMDMs treated with different agents (1 μM, 12 h). (**B**) Quantification of PD-L1 mean fluorescence intensity (MFI) corresponding to (**A**) (n = 3). (**C**) Western blot analysis of PD-L1 protein levels in BMDMs following treatment with different agents. (**D**) Analysis of intracellular drug uptake efficiency in macrophages treated with L-DOX, DOX, PTX, DTX, Nab-PTX, or L-PTX (n = 5). (**E**,**F**) Representative flow cytometry histograms showing PD-L1 expression on BMDMs treated with L-DOX at increasing concentrations (**E**) or for increasing durations (**F**). (**G**,**H**) Quantification of PD-L1 MFI corresponding to (**E**) and (**F**), respectively (n = 3). (**I**,**J**) Western blot analysis of PD-L1 protein levels in macrophages treated with L-DOX at increasing concentrations (**I**) or for increasing durations (**J**). Densitometric analyses corresponding to the Western blots in (**C**,**I**,**J**) are provided in Fig. S2F–H. (**K**,**L**) Representative flow cytometry histograms and quantification of PD-L1 MFI in primary hMDMs treated with control, DOX, or L-DOX (1 μM, 24 h; n = 3). (**M**,**N**) Representative flow cytometry histograms and quantification of PD-L1 MFI in BMDMs, BMDCs, EO771 cells, and 4T1 cells treated with or without L-DOX (1 μM, 12 h; n = 3). Samples in (**M**,**N**) were stained and acquired in parallel under the same flow cytometry settings. Statistical tests: one-way ANOVA with Tukey’s correction (**B**,**L**); two-way ANOVA with Sidak’s correction for indicated comparisons (**D**,**N**); one-way ANOVA with Dunnett’s correction (**G**,**H**). *p < 0.05, **p < 0.01, ***p < 0.001, ****p < 0.0001. ns, not significant (*p* ≥ 0.05). Abbreviations: L-DOX, liposomal doxorubicin; DOX, doxorubicin; PTX, paclitaxel; DTX, docetaxel; Nab-PTX, nanoparticle albumin-bound paclitaxel; L-PTX, liposomal paclitaxel; PD-L1, programmed death-ligand 1; BMDM, bone marrow-derived macrophage; hMDM, human monocyte-derived macrophage; BMDC, bone marrow-derived dendritic cell; MFI, mean fluorescence intensity.

To identify chemotherapeutic drivers of macrophage PD-L1 induction during breast cancer NAC, we compared L-DOX, DOX, PTX, DTX, Nab-PTX, and L-PTX in BMDMs [3]. Flow cytometry and Western blotting showed that L-DOX induced the strongest PD-L1 upregulation among all agents (Fig. 2A–C and Fig. S2A). L-PTX also induced higher PD-L1 expression than non-liposomal PTX, suggesting that liposomal formulation may enhance macrophage targeting. Consistently, fluorescence-based uptake assays showed greater macrophage accumulation of L-DOX than DOX in a dose-dependent manner (Fig. 2D).

We next characterized the dose- and time-dependent regulation of PD-L1 expression by L-DOX. Both flow cytometry and Western blot analysis revealed a clear dose- and time-dependent increase in PD-L1 following L-DOX treatment (Fig. 2E–J and Fig. S2B,C). Cell viability assays further showed that macrophage viability was significantly reduced when L-DOX exceeded 1 μM or when treatment duration extended beyond 24 h (Fig. S2D,E). Densitometric analyses of the Western blots shown in Fig. 2C,I,J confirmed these findings (Fig. S2F–H). After confirming successful differentiation of BMDMs as live CD11b^+^F4/80^+^ macrophages and primary hMDMs as live CD45^+^CD11b^+^CD64^+^ macrophages by flow cytometry (Fig. S2I,J), we treated hMDMs with equimolar DOX or L-DOX and found that both increased PD-L1 expression, with a stronger effect observed for L-DOX (Fig. 2K,L). Across BMDMs, BMDCs, EO771 cells, and 4T1 cells, L-DOX induced PD-L1 most prominently in BMDMs, with weaker changes in BMDCs and tumor cells (Fig. 2M,N), supporting a preferential macrophage PD-L1 response *in vitro*.

### 3.3 L-DOX Activates DNA Damage Signaling to Induce PD-L1 Expression

**Figure 3:**
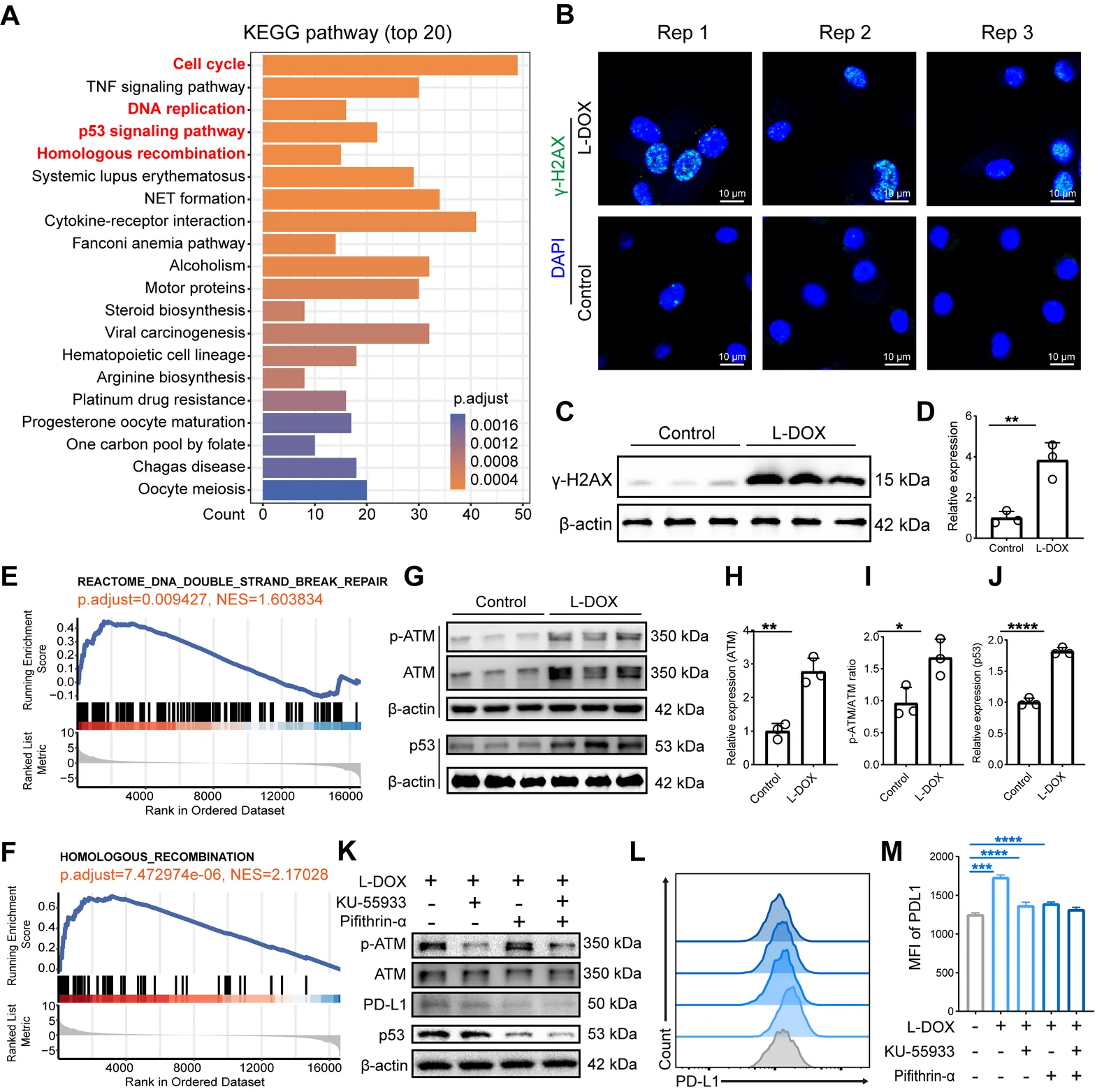
**L-DOX activates DNA damage signaling to induce PD-L1 expression.** (**A**) Top 20 KEGG pathways enriched in RNA-seq of L-DOX-treated macrophages (ranked by *p*-value). (**B**) Immunofluorescence staining of γ-H2A.X (green) and DAPI (blue) in control and L-DOX-treated macrophages. Images are representative of three independent experiments (scale bar = 10 μm). (**C**,**D**) Western blot and densitometric analysis showing increased γ-H2A.X levels after L-DOX treatment (n = 3). (**E**,**F**) GSEA plots showing enrichment of DNA double-strand break repair (**E**) and homologous recombination (**F**) pathways after L-DOX exposure (NES > 0 indicates activation; *p* < 0.05). (**G**) Western blot showing p-ATM, ATM, and p53 protein levels in control and L-DOX-treated macrophages. (**H**–**J**) Densitometric analyses of total ATM (**H**), p-ATM/ATM ratio (**I**), and p53 (**J**) (n = 3). (**K**) Western blot showing PD-L1 protein levels in macrophages treated with L-DOX alone or co-treated with ATM inhibitor (KU-55933) or p53 inhibitor (Pifithrin-α). (**L**) Representative flow cytometry histograms of PD-L1 expression under indicated treatments. (**M**) Quantification of PD-L1 MFI corresponding to (**L**) (n = 3). Statistical tests: two-tailed unpaired Student’s *t*-test (**D**,**H**–**J**); one-way ANOVA with Sidak’s correction for indicated comparisons (**M**). *p < 0.05, **p < 0.01, ***p < 0.001, ****p < 0.0001. Abbreviations: L-DOX, liposomal doxorubicin; PD-L1, programmed death-ligand 1; KEGG, Kyoto encyclopedia of genes and genomes; GSEA, gene set enrichment analysis; NES, normalized enrichment score; ATM, ataxia-telangiectasia mutated; DAPI, 4′,6-diamidino-2-phenylindole; MFI, mean fluorescence intensity.

To investigate the molecular mechanisms underlying L-DOX-induced PD-L1 upregulation, we performed RNA-seq on macrophages treated with L-DOX (1 μM, 24 h). KEGG analysis showed significant enrichment of DNA damage-related pathways, including cell cycle, DNA replication, homologous recombination, and p53 signaling pathway (Fig. 3A and Fig. S3A). GO analysis produced similar results (Fig. S3B), suggesting that L-DOX triggers a DNA damage response in macrophages.

We validated this observation by assessing γ-H2A.X, a canonical marker of DNA double-strand breaks (DSB), using immunofluorescence (Fig. 3B and Fig. S3C) and Western blotting (Fig. 3C,D). Both assays confirmed DSB formation after L-DOX exposure, in line with DOX’s mechanism as a topoisomerase II inhibitor [22]. GSEA further suggested enrichment of DNA double-strand break repair, homologous recombination, and p53 signaling pathways after L-DOX treatment (Fig. 3E,F and Fig. S3D–F). L-DOX-treated macrophages also showed transcriptional signatures of SASP and cellular senescence (Fig. S3G–I), which were validated by β-galactosidase staining (Fig. S3J,K) and increased p21 levels (Fig. S3L,M).

Because γ-H2A.X accumulation indicated DNA double-strand breaks, which activate ATM-dependent DNA damage signaling and downstream p53 responses [23], we examined the ATM-p53 axis. Western blotting revealed increased ATM phosphorylation (p-ATM/ATM), total ATM, and p53 expression in L-DOX-treated macrophages (Fig. 3G–J), corroborating pathway activation. To test causality, macrophages were co-treated with L-DOX and either an ATM inhibitor (KU-55933) or a p53 inhibitor (Pifithrin-α). Both inhibitors substantially attenuated L-DOX-induced PD-L1 upregulation (Fig. 3K–M).

### 3.4 L-DOX Upregulates PD-L1 through the cGAS-STING-p65-Dependent NF-κB Signaling Axis

Having established the ATM-p53-mediated DNA damage response as an upstream requirement for PD-L1 induction, we next explored how DNA damage signals regulate PD-L1 transcription. Genotoxic stress has been reported to activate innate immune sensing pathways, particularly the cGAS-STING axis, which is implicated in immune checkpoint regulation [24,25]. We therefore examined whether cGAS-STING signaling mediates L-DOX-induced PD-L1 upregulation in macrophages.

**Figure 4:**
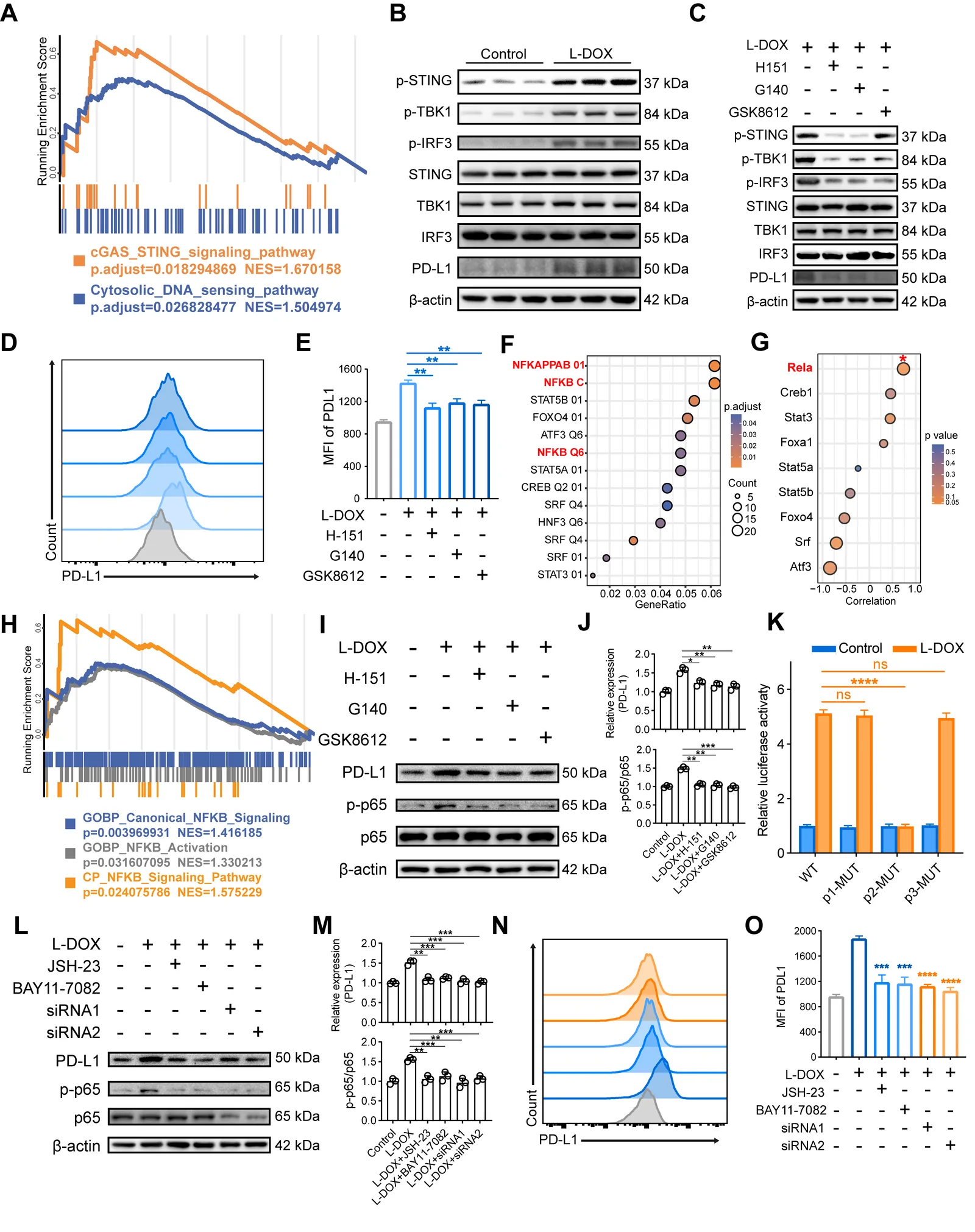
**L-DOX upregulates PD-L1 through the cGAS-STING-p65-dependent NF-κB signaling axis.** (**A**) GSEA plot showing activation of cGAS-STING pathways after L-DOX treatment. (**B**) Western blot of STING, p-STING, TBK1, p-TBK1, IRF3, p-IRF3, and PD-L1 after L-DOX. (**C**) Western blot showing the effects of pharmacological inhibition at different nodes of the cGAS-STING pathway using G140, H-151, and GSK8612 on L-DOX-induced signaling and PD-L1 expression. (**D**) Representative flow cytometry histograms showing PD-L1 expression in BMDMs treated with L-DOX in the presence or absence of the indicated cGAS-STING pathway inhibitors. (**E**) Quantification of PD-L1 mean fluorescence intensity (MFI) corresponding to (**D**) (n = 3). (**F**) TF target enrichment analysis (dot size = gene count; color = *p*-value). (**G**) Correlation analysis between enriched TFs and PD-L1 expression (dot size = correlation coefficient; color = *p*-value). (**H**) GSEA plot showing NF-κB pathway activation after L-DOX treatment. (**I**) Representative Western blot showing the effects of H-151, G140, and GSK8612 on L-DOX-induced p65 phosphorylation and PD-L1 expression. (**J**) Densitometric quantification of p-p65 relative to total p65 and PD-L1 relative to β-actin corresponding to (**I**) (n = 3). (**K**) Dual-luciferase reporter assay comparing WT and mutant (p1–p3) PD-L1 promoters after L-DOX treatment. (**L**) Representative Western blot showing the effects of the NF-κB inhibitors JSH-23 and BAY 11-7082 or p65-targeting siRNA on L-DOX-induced p65 activation and PD-L1 expression. (**M**) Densitometric quantification of p-p65 relative to total p65 and PD-L1 relative to β-actin corresponding to (**L**) (n = 3). (**N**) Representative flow cytometry histograms showing PD-L1 expression following pharmacological NF-κB inhibition or siRNA-mediated p65 knockdown in L-DOX-treated BMDMs. (**O**) Quantification of PD-L1 MFI corresponding to (**N**) (n = 3). Statistical tests: one-way ANOVA with Sidak’s correction for indicated comparisons (**E**,**J**,**M**,**O**); two-way ANOVA with Sidak’s correction (**K**); hypergeometric test (**F**); Pearson correlation test (**G**). *p < 0.05, **p < 0.01, ***p < 0.001, ****p < 0.0001. ns, not significant (*p* ≥ 0.05). Abbreviations: L-DOX, liposomal doxorubicin; PD-L1, programmed death-ligand 1; cGAS, cyclic GMP-AMP synthase; STING, stimulator of interferon genes; TBK1, TANK-binding kinase 1; IRF3, interferon regulatory factor 3; NF-κB, nuclear factor kappa B; GSEA, gene set enrichment analysis; TF, transcription factor; WT, wild-type; MUT, mutant; siRNA, small interfering RNA; MFI, mean fluorescence intensity.

GSEA revealed enrichment of cGAS-STING signaling in L-DOX-treated macrophages (Fig. 4A and Fig. S4A). Western blotting showed increased phosphorylation of STING, TBK1, and IRF3 alongside elevated PD-L1 protein (Fig. 4B and Fig. S4B–E). Pharmacological inhibition of the cGAS-STING pathway using H-151 (STING inhibitor), GSK8612 (TBK1 inhibitor acting downstream of STING), or G140 (cGAS inhibitor) markedly reduced L-DOX-induced PD-L1 elevation (Fig. 4C–E and Fig. S4F).

cGAS-STING activates two major transcriptional branches mediated by IRF3 and NF-κB [26]. Transcription factor (TF) target enrichment analysis preferentially implicated RELA/p65-associated NF-κB signaling rather than IRF family members (Fig. 4F). Correlation analysis revealed a strong positive association between PD-L1 and *Rela* (encoding NF-κB p65) (Fig. 4G), and GSEA indicated robust NF-κB pathway activation after L-DOX treatment (Fig. 4H). Consistent with this finding, inhibition of cGAS-STING signaling reduced p65 phosphorylation and PD-L1 expression, placing p65-dependent NF-κB signaling downstream of cGAS-STING activation in L-DOX-treated macrophages (Fig. 4I,J). ChIP-qPCR and dual-luciferase reporter assays, guided by JASPAR-predicted NF-κB binding sites (p1–p3) in the PD-L1 promoter, demonstrated direct binding of p65 to a specific NF-κB-responsive element and consequent transcriptional activation (Fig. 4K and Fig. S4G,H). Pharmacological NF-κB inhibition (JSH-23, BAY 11-7082) or siRNA-mediated p65 knockdown reduced p65 activation and reversed PD-L1 induction at the protein level (Fig. 4L,M and Fig. S4I,J), and flow cytometry further confirmed reduced PD-L1 expression under these conditions (Fig. 4N,O and Fig. S4K).

To further validate this mechanism in a human macrophage model, we treated primary hMDMs with L-DOX in the presence or absence of H-151 or JSH-23. Both inhibitors attenuated L-DOX-induced PD-L1 upregulation, as measured by PD-L1 MFI, whereas inhibitor treatment alone had minimal effects on basal PD-L1 expression (Fig. S4L,M). These results support that L-DOX activates cGAS-STING, engaging p65-dependent NF-κB signaling to drive PD-L1 transcription in macrophages.

### 3.5 Anti-PD-L1 Promotes a Pro-Inflammatory Shift in Macrophage Phenotypes

PD-L1 expression in macrophages can contribute to immune suppression [27], whereas anti-PD-L1 therapy can block checkpoint signaling and promote pro-inflammatory macrophage phenotypes [28,29]. We therefore hypothesized that, in the context of L-DOX-induced PD-L1 upregulation, addition of anti-PD-L1 would simultaneously block checkpoint signaling and reprogram macrophages to amplify CD8^+^ T-cell activation and antitumor immunity.

**Figure 5:**
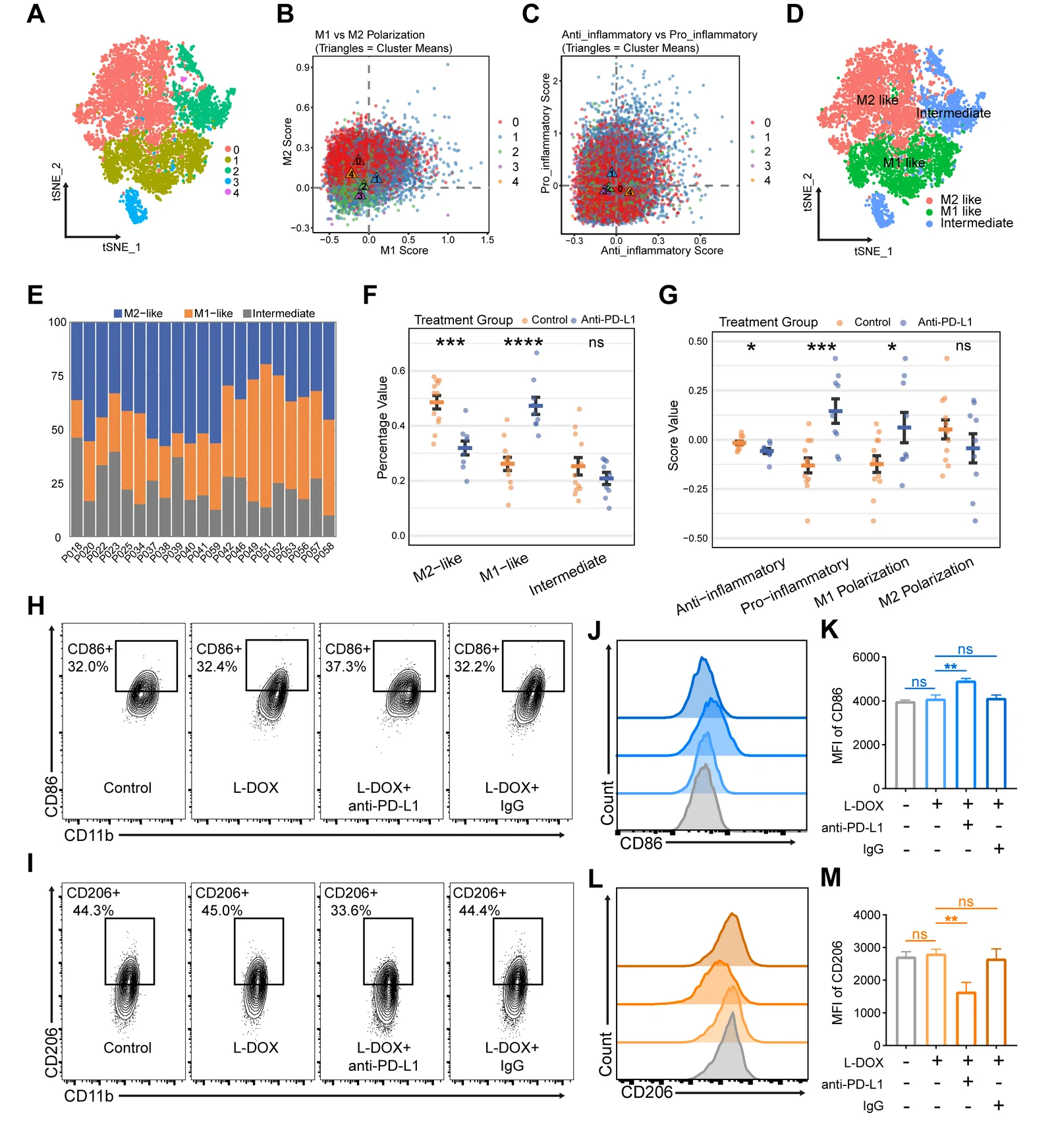
**Anti-PD-L1 promotes a pro-inflammatory shift in macrophage phenotypes.** (**A**) t-SNE clustering of monocyte-macrophages from GSE266919. (**B**,**C**) AddModuleScore analysis of M1/M2-like signatures (**B**) and pro-/anti-inflammatory signatures (**C**); triangles indicate cluster averages. (**D**) t-SNE visualization of macrophage subsets classified as M1-like/pro-inflammatory, M2-like/immunoregulatory, or intermediate based on relative signature enrichment scores. (**E**) Stacked bar chart showing macrophage subset proportions across patient samples. (**F**) Statistical comparison of macrophage subset proportions between anti-PD-L1 and control groups. (**G**) Comparison of pro-inflammatory and M1 gene set scores between treatment groups. (**H**,**I**) Flow cytometry quantification of CD86^+^ F4/80^+^ CD11b^+^ (**H**) and CD206^+^ F4/80^+^ CD11b^+^ (**I**) macrophages under different treatments. (**J**) Representative flow cytometry histograms showing CD86 expression in F4/80^+^CD11b^+^ macrophages under the indicated treatments. (**K**) Quantification of CD86 mean fluorescence intensity (MFI) corresponding to (**J**) (n = 3). (**L**) Representative flow cytometry histograms showing CD206 expression in F4/80^+^CD11b^+^ macrophages under the indicated treatments. (**M**) Quantification of CD206 MFI corresponding to (**L**) (n = 3). Statistical tests: Welch’s *t*-test (**F**,**G**); one-way ANOVA with Sidak’s correction for indicated comparisons (**K**,**M**). *p < 0.05, **p < 0.01, ***p < 0.001, ****p < 0.0001. ns, not significant (*p* ≥ 0.05). Abbreviations: PD-L1, programmed death-ligand 1; L-DOX, liposomal doxorubicin; GSE, gene expression omnibus series; t-SNE, t-distributed stochastic neighbor embedding; M1, M1-like/pro-inflammatory macrophage phenotype; M2, M2-like/immunoregulatory macrophage phenotype; IgG, immunoglobulin G; MFI, mean fluorescence intensity.

Analysis of the scRNA-seq dataset from patients treated with chemotherapy plus anti-PD-L1 (GSE266919) revealed distinct monocyte-macrophage subsets after clustering (Fig. 5A and Fig. S5A–C). Using AddModuleScore, we computed M1/M2-like and pro-/anti-inflammatory scores and classified macrophages into M1-like/pro-inflammatory, M2-like/immunoregulatory, and intermediate subgroups based on relative signature enrichment (Fig. 5B–D). A marker heatmap across the same macrophage clusters further showed that CD274 expression was relatively high in clusters 0 and 1, but these two clusters displayed distinct marker profiles. Cluster 0 exhibited higher expression of several M2-like/immunoregulatory markers, whereas cluster 1 showed stronger M1-like/pro-inflammatory marker expression, suggesting heterogeneity among PD-L1-high macrophage clusters (Fig. S5D). Compared with controls, anti-PD-L1 treatment increased the proportion of M1-like/pro-inflammatory macrophages and reduced the proportion of M2-like/immunoregulatory macrophages (Fig. 5E,F), with enrichment of pro-inflammatory and M1-like gene signatures (Fig. 5G). Transcriptomic data from GSE116564 corroborated these shifts, showing increased M1-like markers and reduced M2-like markers after anti-PD-L1 treatment (Fig. S5E).

*In vitro* flow cytometry confirmed that L-DOX alone did not substantially change proportions of CD86^+^ or CD206^+^ macrophages, whereas the combination of L-DOX and anti-PD-L1 significantly increased CD86^+^ cells and decreased CD206^+^ cells (Fig. 5H,I). Corresponding changes in CD86 and CD206 MFI supported phenotype switching toward an M1-like state (Fig. 5J–M). Morphologically, macrophages treated with the combination acquired a rounded appearance consistent with a pro-inflammatory macrophage phenotype (Fig. S5F).

### 3.6 Combining Anti-PD-L1 with L-DOX Alleviates Macrophage-Mediated Suppression of CD8^+^ T Cells

**Figure 6:**
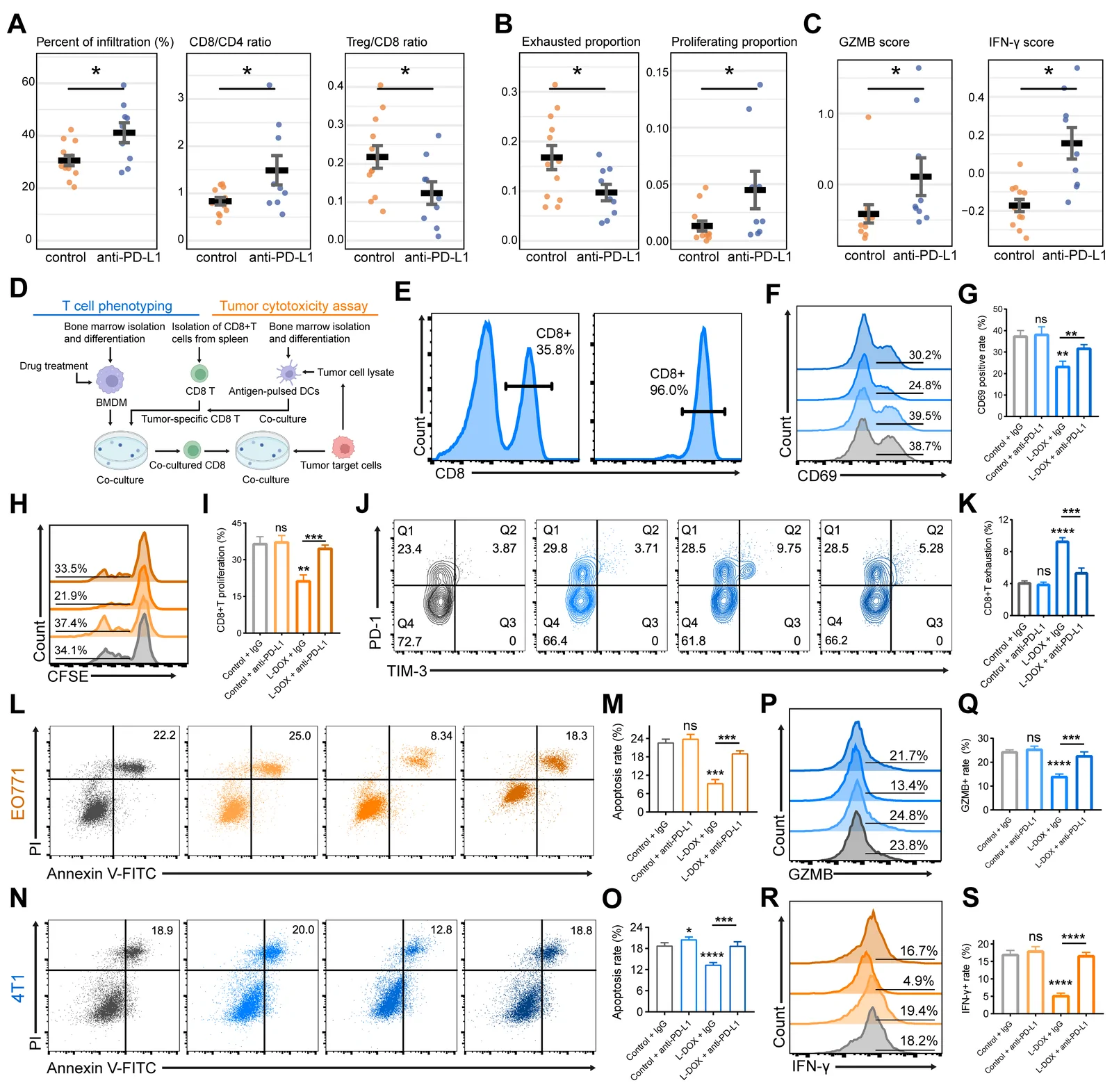
**PD-L1 blockade reverses L-DOX-conditioned macrophage-mediated suppression of CD8^+^ T cells.** (**A**) Effects of combined anti-PD-L1 therapy on T cell features in GSE266919: CD8^+^ infiltration (% of immune cells), CD8/CD4 ratio, and Treg/CD8 ratio. (**B**) CD8^+^ T cell subset features: exhaustion and proliferation proportions. (**C**) AddModuleScore for GZMB and IFN-γ expression in CD8^+^ T cells. (**D**) Schematic of co-culture and cytotoxicity assay. Macrophages were assigned to four treatment conditions: Control + IgG, Control + anti-PD-L1, L-DOX + IgG, and L-DOX + anti-PD-L1. (**E**) Purity check of CD8^+^ T cells before/after magnetic sorting. (**F**) Representative flow cytometry histograms showing CD69 expression in CD8^+^ T cells under the indicated co-culture conditions. (**G**) Quantification of CD69 mean fluorescence intensity corresponding to (**F**) (n = 3). (**H**) Representative CFSE dilution profiles showing CD8^+^ T-cell proliferation under the indicated co-culture conditions. (**I**) Quantification of CD8^+^ T-cell proliferation corresponding to (**H**) (n = 3). (**J**) Representative flow cytometry contour plots showing PD-1^+^TIM-3^+^ exhausted CD8^+^ T cells under the indicated co-culture conditions. (**K**) Quantification of the proportion of PD-1^+^TIM-3^+^ cells among CD8^+^ T cells corresponding to (**J**) (n = 3). (**L**) Representative flow cytometry plots showing the cytotoxicity of tumor-specific CD8^+^ T cells against EO771 target cells. (**M**) Quantification of EO771 target-cell killing corresponding to (**L**) (n = 3). (**N**) Representative flow cytometry plots showing the cytotoxicity of tumor-specific CD8^+^ T cells against 4T1 target cells. (**O**) Quantification of 4T1 target-cell killing corresponding to (**N**) (n = 3). (**P**) Representative flow cytometry histograms showing GZMB expression in CD8^+^ T cells under the indicated co-culture conditions. (**Q**) Quantification of GZMB mean fluorescence intensity corresponding to (**P**) (n = 3). (**R**) Representative flow cytometry histograms showing IFN-γ expression in CD8^+^ T cells under the indicated co-culture conditions. (**S**) Quantification of IFN-γ mean fluorescence intensity corresponding to (**R**) (n = 3). Statistical tests: Welch’s *t*-test (**A**–**C**); one-way ANOVA with Sidak’s correction for indicated pre-specified pairwise comparisons (**G**,**I**,**K**,**M**,**O**,**Q**,**S**). *p < 0.05, **p < 0.01, ***p < 0.001, ****p < 0.0001. ns, not significant (*p* ≥ 0.05). Abbreviations: L-DOX, liposomal doxorubicin; PD-L1, programmed death-ligand 1; PD-1, programmed cell death protein 1; GSE, gene expression omnibus series; Treg, regulatory T cell; BMDM, bone marrow-derived macrophage; BMDC, bone marrow-derived dendritic cell; CFSE, carboxyfluorescein succinimidyl ester; IgG, immunoglobulin G; MFI, mean fluorescence intensity.

We next determined whether the combination therapy alleviates CD8^+^ T cell suppression and enhances antitumor immunity. Using the GSE266919 dataset, we analyzed CD8^+^ and CD4^+^ T cell subsets. After clustering and visualization (Fig. S6A–D), marker analysis (Fig. S6E,F) revealed that anti-PD-L1 treatment increased overall CD8^+^ T cell infiltration, elevated the CD8/CD4 ratio, and reduced the Treg/CD8^+^ T cell ratio (Fig. 6A). Within CD8^+^ T cell subsets, anti-PD-L1 decreased exhaustion-associated signatures while expanding proliferative populations (Fig. 6B). Moreover, the expression of key cytotoxic effector molecules granzyme B (GZMB) and interferon-γ (IFN-γ) was significantly increased following anti-PD-L1 treatment (Fig. 6C and Fig. S6G), indicating enhanced cytotoxic potential.

To directly assess whether PD-L1 blockade specifically reverses L-DOX-conditioned macrophage-mediated immunosuppression, we redesigned the co-culture assay to include four groups: Control + IgG, Control + anti-PD-L1, L-DOX + IgG, and L-DOX + anti-PD-L1 (Fig. 6D). CD8^+^ T cells were magnetically sorted from mouse spleens (Fig. 6E), activated, and co-cultured with macrophages subjected to the indicated treatments. Compared with Control + IgG, Control + anti-PD-L1 produced minimal changes in CD8^+^ T-cell activation. In contrast, L-DOX + IgG markedly suppressed CD8^+^ T-cell activation, as indicated by reduced CD69 and CD25 expression, whereas L-DOX + anti-PD-L1 restored T-cell activation (Fig. 6F,G and Fig. S6H,I).

Similarly, Control + anti-PD-L1 had limited effects on CD8^+^ T-cell proliferation and exhaustion, whereas L-DOX + IgG reduced CFSE dilution and increased the proportion of exhausted PD-1^+^TIM-3^+^ CD8^+^ T cells. These effects were reversed by L-DOX + anti-PD-L1 (Fig. 6H–K). In tumor-specific cytotoxicity assays using BMDCs pulsed with tumor lysates (Fig. S6J), L-DOX + IgG significantly impaired CD8^+^ T-cell-mediated killing of EO771 and 4T1 cells, whereas L-DOX + anti-PD-L1 restored cytotoxicity (Fig. 6L–O and Fig. S6K,L). Consistently, GZMB and IFN-γ expression in CD8^+^ T cells was reduced by L-DOX + IgG and restored by L-DOX + anti-PD-L1 (Fig. 6P–S). These results indicate that PD-L1 blockade primarily counteracts L-DOX-conditioned macrophage-mediated T-cell suppression rather than acting as a strong standalone enhancer in this co-culture system.

### 3.7 Combined L-DOX and Anti-PD-L1 Therapy Achieves Superior Antitumor Efficacy In Vivo

**Figure 7:**
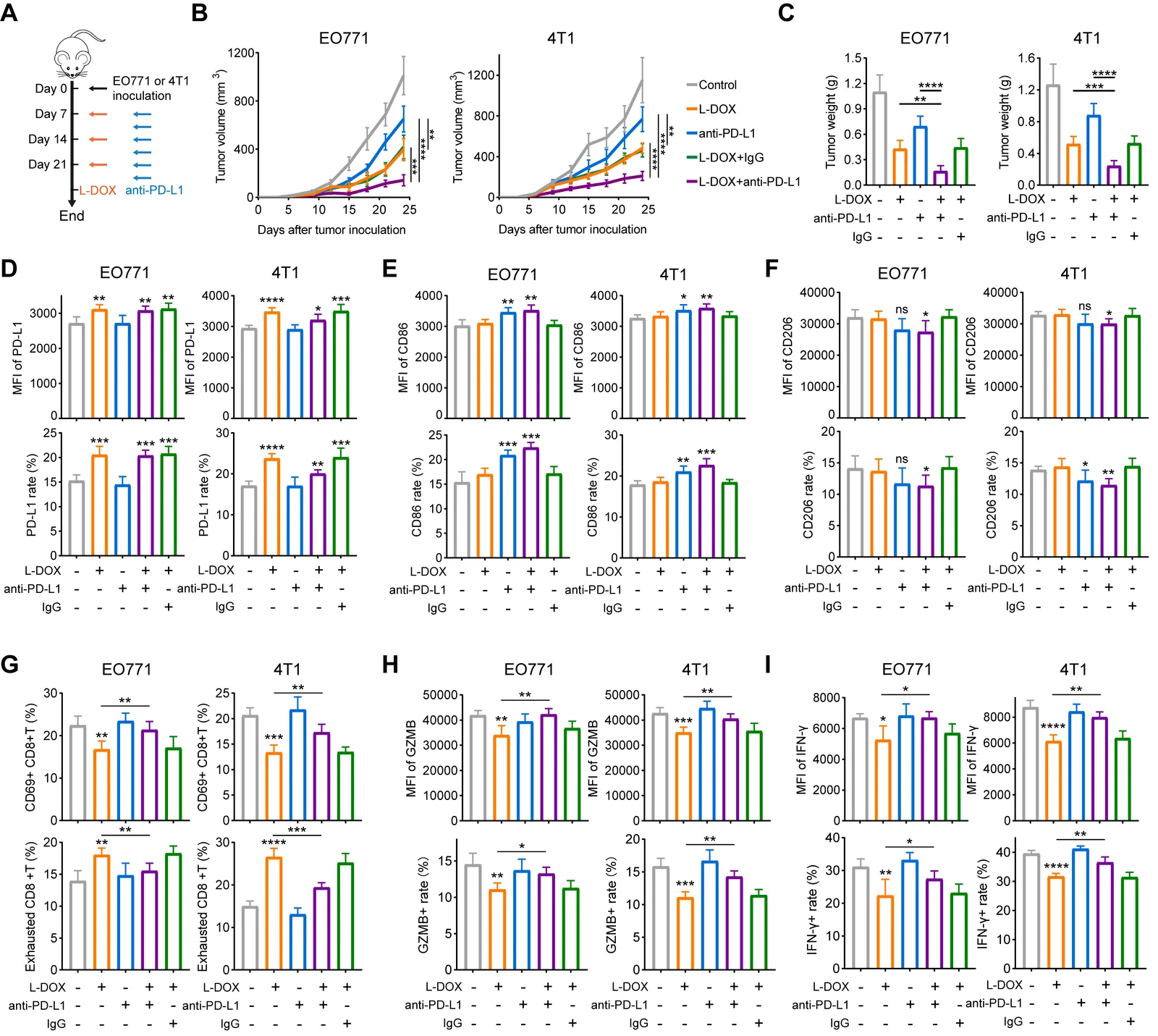
**Combined L-DOX and anti-PD-L1 therapy achieves superior antitumor efficacy *in vivo*.** (**A**) Schematic diagram of the treatment regimen for mice inoculated with EO771 or 4T1 tumors. (**B**) Tumor growth curves for EO771 (left) and 4T1 (right) models during treatment (n = 5). (**C**) Tumor weights at endpoint for EO771 (left) and 4T1 (right) models (n = 5). (**D**) Flow cytometry analysis of PD-L1 MFI (top) and percentage of PD-L1^+^ cells (bottom) among tumor-infiltrating macrophages (CD45^+^CD11b^+^F4/80^+^). (**E**) Flow cytometric quantification of CD86 MFI and the percentage of CD86^+^ cells among tumor-infiltrating macrophages under the indicated treatments. (**F**) Flow cytometric quantification of CD206 MFI and the percentage of CD206^+^ cells among tumor-infiltrating macrophages under the indicated treatments. (**G**) Percentage of activated (CD69^+^) and exhausted (PD-1^+^TIM-3^+^) tumor-infiltrating CD8^+^ T cells. (**H**) Flow cytometric quantification of GZMB MFI and the percentage of GZMB^+^ cells among tumor-infiltrating CD8^+^ T cells. (**I**) Flow cytometric quantification of IFN-γ MFI and the percentage of IFN-γ^+^ cells among tumor-infiltrating CD8^+^ T cells. Statistical tests: two-way repeated-measures ANOVA with Sidak’s correction (**B**); one-way ANOVA with Sidak’s correction for indicated comparisons (**C**–**I**). *p < 0.05, **p < 0.01, ***p < 0.001, ****p < 0.0001. ns, not significant (*p* ≥ 0.05). Abbreviations: L-DOX, liposomal doxorubicin; PD-L1, programmed death-ligand 1; PD-1, programmed cell death protein 1; TAM, tumor-associated macrophage; IgG, immunoglobulin G; MFI, mean fluorescence intensity.

We evaluated the efficacy of the combination treatment in two TNBC mouse models, EO771 and 4T1 (Fig. 7A). Both L-DOX and anti-PD-L1 delayed tumor growth; however, the combination therapy resulted in significantly enhanced tumor suppression (Fig. 7B and Fig. S7A). At the endpoint, tumor weights were lowest in the combination group and were significantly reduced compared with either single-agent treatment (Fig. 7C).

Flow cytometric analysis (gating strategy in Fig. S7B) showed that L-DOX did not significantly increase total macrophage infiltration in either EO771 or 4T1 tumors (Fig. S7C). L-DOX treatment modestly increased the percentage of tumor-infiltrating CD8^+^ T cells, whereas anti-PD-L1 did not further increase CD8^+^ T-cell infiltration (Fig. S7D). Despite the limited change in macrophage abundance, L-DOX robustly upregulated PD-L1 expression on tumor-infiltrating macrophages, in agreement with our *in vitro* findings (Fig. 7D).

To assess whether tumor-cell PD-L1 contributed to this response, we further compared PD-L1 expression in CD45^−^EpCAM^+^ tumor cells and CD45^+^CD11b^+^F4/80^+^ TAMs from control and L-DOX-treated EO771 and 4T1 tumors. Anti-PD-L1-treated groups were excluded from this comparison because therapeutic antibody binding may interfere with flow cytometric PD-L1 detection. L-DOX markedly increased PD-L1 MFI in TAMs but induced only minor changes in CD45^−^EpCAM^+^ tumor cells (Fig. S7E), supporting a macrophage-dominant PD-L1 response *in vivo*.

Anti-PD-L1 attenuated this L-DOX-associated suppressive macrophage phenotype and promoted a shift toward a pro-inflammatory state, as shown by increased CD86 and reduced CD206 expression (Fig. 7E,F). Notably, L-DOX monotherapy impaired CD8^+^ T-cell activation and increased exhaustion, effects that were largely reversed by combination therapy (Fig. 7G). In line with these functional changes, the expression of GZMB and IFN-γ, which was suppressed by L-DOX alone, was restored upon combined treatment with anti-PD-L1 (Fig. 7H,I). These *in vivo* findings indicate that combining L-DOX with PD-L1 blockade enhances antitumor efficacy and remodels macrophage and CD8^+^ T-cell immune states within the tumor microenvironment.

## 4 Discussion

Our study reveals an underappreciated immunomodulatory function of L-DOX in breast cancer NAC. We show that L-DOX preferentially induces PD-L1 expression in macrophages via DNA damage-associated activation of the cGAS-STING-p65-dependent NF-κB axis, establishing a macrophage-centered PD-L1-high program with suppressive effects on CD8^+^ T-cell function and linking L-DOX treatment to adaptive immune resistance.

The preferential impact of L-DOX on macrophages is likely influenced by its unique pharmacological properties. Liposomal formulations prolong systemic circulation and enhance tumor accumulation, conferring a well-established pharmacokinetic advantage over free drug formulations [30]. In addition, nanoparticle-based therapeutics are known to preferentially distribute to the mononuclear phagocyte system, with TAMs exhibiting particularly efficient uptake [31,32]. Such selective accumulation provides a plausible biological basis for our observation that macrophages represent the predominant cellular source of PD-L1 upregulation following L-DOX exposure, distinguishing L-DOX from other chemotherapeutic agents with less pronounced myeloid targeting.

BMDMs provide a controlled model for studying macrophage-intrinsic responses to L-DOX, but they cannot fully recapitulate the heterogeneity of TAMs shaped by tumor, stromal, immune, and metabolic cues *in vivo*. We therefore interpret the BMDM data as mechanistic evidence rather than a complete reproduction of TAM biology. To improve translational relevance, we validated key findings in primary hMDMs, compared macrophages with dendritic cells and tumor cells *in vitro*, and analyzed TAMs and CD45^−^EpCAM^+^ tumor cells *in vivo*. Together, these data support a macrophage-dominant PD-L1 response to L-DOX while acknowledging the limitations of simplified macrophage models.

Our results confirm that L-DOX induces DNA damage and activates the cGAS-STING pathway in macrophages, consistent with previous work describing anthracycline-triggered innate immune sensing [33]. Previous studies have shown that activation of the cGAS-STING pathway initiates two parallel transcriptional branches mediated by IRF3 and NF-κB [26,34]. Importantly, in myeloid cells such as macrophages, activated STING can preferentially engage NF-κB-associated programs [35,36], a signaling context closely linked to immune checkpoint regulation. Within this framework, although L-DOX increased IRF3 phosphorylation and type I interferon-related signatures in our system, multiple complementary lines of evidence indicate that RELA/p65-dependent NF-κB signaling is the principal transcriptional effector driving PD-L1 upregulation in macrophages. This interpretation aligns with previous reports demonstrating direct binding of NF-κB to the PD-L1 promoter and enhanced transcriptional activity [37]. IRF3 activation likely reflects a parallel interferon response (e.g., SASP/chemokines) but was not required for PD-L1 transcription in our assays. Overall, these findings underscore PD-L1 induction as a key immunomodulatory consequence of L-DOX and provide a mechanistic rationale for combining L-DOX with PD-L1 blockade to mitigate therapy-associated immune suppression.

PD-L1-high TAMs can mediate immune suppression within the tumor microenvironment by restraining CD8^+^ T cell activation, proliferation, and cytotoxicity through engagement of the PD-1/PD-L1 axis [38]. Our results demonstrate that although L-DOX exerts direct cytotoxic effects on tumor cells, it simultaneously drives macrophages toward a PD-L1-high state with immunosuppressive features, thereby limiting optimal antitumor T cell responses. This finding underscores an important principle: chemotherapy can induce an adaptive, therapy-associated layer of immune suppression that constrains its own efficacy. In this context, PD-L1 blockade not only relieves inhibitory signaling in T cells but also promotes functional reprogramming of macrophages toward a pro-inflammatory (M1-like) phenotype. Previous studies have shown that anti-PD-L1 antibodies can directly modulate macrophage polarization [28,29,39,40], and our data suggest that this effect may be particularly pronounced in the setting of L-DOX-induced PD-L1 upregulation. Thus, TAM-associated PD-L1 emerges as a key immunological node underlying the enhanced antitumor efficacy observed with L-DOX and PD-L1 blockade.

These findings have translational implications. L-DOX-induced PD-L1 expression in macrophages represents a dynamic, chemotherapy-triggered checkpoint that may help identify tumors more likely to benefit from ICB. PD-L1 expression in immune cells, particularly myeloid populations, can better reflect the immunosuppressive state of the tumor microenvironment and has been associated with responses to immune checkpoint inhibitors in some settings [41]. Clinical studies in TNBC also support the predictive value of immune-cell PD-L1 for chemoimmunotherapy benefit [12,42]. Monitoring TAM PD-L1 after L-DOX treatment may therefore help guide patient stratification and treatment sequencing.

Several limitations should be acknowledged. Although our findings were validated across multiple experimental systems, including primary human monocyte-derived macrophages and *in vivo* tumor models, matched clinical samples from patients receiving L-DOX-based treatment were not available. In addition, BMDMs and hMDMs remain simplified macrophage models and cannot fully recapitulate the heterogeneity of TAMs within the tumor microenvironment. Future studies using paired clinical specimens, high-resolution single-cell and spatial profiling, and systematic pharmacokinetic/pharmacodynamic analyses will be important to further define macrophage-specific regulatory programs *in vivo* and strengthen the translational interpretation of this therapeutic strategy.

In summary, our study demonstrates that L-DOX induces a macrophage-centered PD-L1-high state with immunosuppressive features, which constrains antitumor immunity but can be attenuated by PD-L1 blockade. These findings provide a mechanistic and immunological rationale for integrating PD-L1 inhibitors with liposomal anthracycline-based regimens and support the development of TAM-focused biomarkers to optimize neoadjuvant therapy for TNBC.

## 5 Conclusion

L-DOX promotes a PD-L1-high macrophage phenotype that suppresses antitumor immunity, whereas PD-L1 blockade attenuates this effect and supports a potential chemoimmunotherapy strategy for TNBC.

## Data Availability

The public datasets analyzed in this study are available from the Gene Expression Omnibus under accession numbers GSE28583, GSE28826, GSE232764, GSE260989, GSE149479, GSE133683, GSE116564, GSE279219, GSE266919 and GSE191246. The RNA-seq data generated in this study have been deposited in the China National Center for Bioinformation under accession number PRJCA052710. Other data supporting the findings of this study are available within the article and Supplementary Materials, or from the corresponding author upon reasonable request.

## Supplementary Materials

The supplementary material is available online at https://www.techscience.com/doi/10.32604/or.2026.087138/s1. References [43,44] are cited in the Supplementary Materials.

## Abbreviations

TNBC	triple-negative breast cancer
NAC	neoadjuvant chemotherapy
TME	tumor microenvironment
DOX	doxorubicin
L-DOX	liposomal doxorubicin
PD-L1	programmed death-ligand 1
PD-1	programmed cell death protein 1
TAMs	tumor-associated macrophages
ICB	immune checkpoint blockade
BMDMs	bone marrow-derived macrophages
hMDMs	human monocyte-derived macrophages
BMDCs	bone marrow-derived dendritic cells
Treg	regulatory T cell
STR	short tandem repeat
RNA-seq	RNA sequencing
scRNA-seq	single-cell RNA sequencing
GEO	gene expression omnibus
CNCB	China national center for bioinformation
PCA	principal component analysis
t-SNE	t-distributed stochastic neighbor embedding
UMI	unique molecular identifier
KEGG	Kyoto encyclopedia of genes and genomes
GO	gene ontology
GSEA	gene set enrichment analysis
MSigDB	molecular signatures database
SASP	senescence-associated secretory phenotype
TF	transcription factor
ATM	ataxia-telangiectasia mutated
cGAS	cyclic GMP-AMP synthase
STING	stimulator of interferon genes
TBK1	TANK-binding kinase 1
IRF3	interferon regulatory factor 3
NF-κB	nuclear factor kappa B
ChIP	chromatin immunoprecipitation
qPCR	quantitative polymerase chain reaction
siRNA	small interfering RNA
PTX	paclitaxel
DTX	docetaxel
Nab-PTX	nanoparticle albumin-bound paclitaxel
L-PTX	liposomal paclitaxel
DSB	double-strand break
CCK-8	cell counting kit-8
CFSE	carboxyfluorescein succinimidyl ester
MFI	mean fluorescence intensity
GZMB	granzyme B
IFN-γ	interferon gamma
FDR	false discovery rate
IgG	immunoglobulin G
