# Supplementary material for: Liposomal Doxorubicin Induces PD-L1-High Tumor-Associated Macrophages and Sensitizes Triple-Negative Breast Cancer to PD-L1 Blockade
Source: Oncol Res. 2026 Sep 14;34(10):28. doi: 10.32604/or.2026.087138 (PMC13591353; doi:10.32604/or.2026.087138)
Supplement: Supplementary file 1 [file OncolRes-34-87138-s001.zip › TSP_OR_87138-s001.docx]

Supplementary Materials

**Figure S1: Quality control and validation of bioinformatics analyses.** (**A**) PCA plot of bulk RNA-seq datasets after ComBat correction. (**B**) Tumor immune-cell infiltration estimated using the quanTIseq and CIBERSORT deconvolution algorithms in the human bulk transcriptomic datasets. (**C**) Tumor immune-cell infiltration estimated using the quanTIseq and CIBERSORT deconvolution algorithms in the murine bulk transcriptomic datasets. (**D**) MCP-counter analysis comparing immune-cell abundance between the treatment and control groups in the human bulk transcriptomic datasets. (**E**) MCP-counter analysis comparing immune-cell abundance between the treatment and control groups in the murine bulk transcriptomic datasets. (**F**) t-SNE visualization of cells from the human TNBC single-cell RNA-seq dataset GSE279219. (**G**) Dot plot showing the expression of canonical marker genes used for cell-type annotation in GSE279219. (**H**) t-SNE visualization of cells from the murine single-cell RNA-seq dataset GSE191246. (**I**) Dot plot showing the expression of canonical marker genes used for cell-type annotation in GSE191246. The marker genes used for cell-type annotation are listed in Table S3. Statistical tests: Spearman correlation test (**C**); two-tailed paired Student’s *t*-test (**D**); two-tailed unpaired Student’s *t*-test (**E**). **p* < 0.05. Abbreviations: PCA, principal component analysis; PC, principal component; RNA-seq, RNA sequencing; scRNA-seq, single-cell RNA sequencing; GSE, gene expression omnibus series; TNBC, triple-negative breast cancer; DOX, doxorubicin; quanTIseq, quantification of the tumor immune contexture from RNA-seq data; CIBERSORT, cell-type identification by estimating relative subsets of rna transcripts; MCP-counter, microenvironment cell populations-counter; t-SNE, t-distributed stochastic neighbor embedding; Treg, regulatory T cell; NK, natural killer; M0, unpolarized macrophage phenotype; M1, M1-like/pro-inflammatory macrophage phenotype; M2, M2-like/immunoregulatory macrophage phenotype.

**Figure S2: L-DOX-induced PD-L1 expression and macrophage characterization.** (**A**) Flow cytometric analysis of the percentage of PD-L1^+^ F4/80^+^ CD11b^+^ macrophages following treatment with different chemotherapeutic agents. (**B**) Flow cytometric quantification of the percentage of PD-L1^+^ cells among F4/80^+^CD11b^+^ BMDMs treated with increasing concentrations of L-DOX. (**C**) Flow cytometric quantification of the percentage of PD-L1^+^ cells among F4/80^+^CD11b^+^ BMDMs treated with L-DOX for increasing durations. (**D**) BMDM viability assessed by CCK-8 assay following treatment with increasing concentrations of L-DOX. (**E**) BMDM viability assessed by CCK-8 assay following L-DOX treatment for increasing durations. (**F**) Densitometric quantification of PD-L1 protein expression corresponding to the Western blot shown in Fig. 2C (n = 3). (**G**) Densitometric quantification of PD-L1 protein expression corresponding to the Western blot shown in Fig. 2I (n = 3). (**H**) Densitometric quantification of PD-L1 protein expression corresponding to the Western blot shown in Fig. 2J (n = 3). (**I**) Representative flow cytometric gating and phenotypic characterization of differentiated BMDMs as live CD11b^+^F4/80^+^ cells. (**J**) Representative flow cytometric gating and phenotypic characterization of differentiated hMDMs as live CD45^+^CD11b^+^CD64^+^ cells. Statistical tests: one-way ANOVA with Sidak’s correction for indicated comparisons (**D**–**F**); one-way ANOVA with Dunnett’s correction versus baseline (**G**,**H**). **p* < 0.05, ***p* < 0.01, ****p* < 0.001, *****p* < 0.0001. ns, not significant (*p* ≥ 0.05). Abbreviations: L-DOX, liposomal doxorubicin; DOX, doxorubicin; PTX, paclitaxel; DTX, docetaxel; Nab-PTX, nanoparticle albumin-bound paclitaxel; L-PTX, liposomal paclitaxel; PD-L1, programmed death-ligand 1; BMDM, bone marrow-derived macrophage; hMDM, human monocyte-derived macrophage; CCK-8, cell counting kit-8; MFI, mean fluorescence intensity.

**Figure S3: L-DOX induces DNA damage and senescence in macrophages.** (**A**) Volcano plot of differentially expressed genes from RNA-seq (n = 4): orange, upregulated (*p* < 0.05, |log_2_ FC| > 1); blue, downregulated (*p* < 0.05, |log_2_ FC| > 1). (**B**) Top 10 GO terms. (**C**) Representative γ-H2A.X immunofluorescence images (scale bar = 10 μm). (**D**) GSEA plot showing enrichment of the homologous recombination pathway in L-DOX-treated BMDMs. (**E**) GSEA plot showing enrichment of the ATM signaling pathway in L-DOX-treated BMDMs. (**F**) GSEA plot showing enrichment of the p53 signaling pathway in L-DOX-treated BMDMs. (**G**) GSEA plot showing enrichment of the senescence-associated secretory phenotype signature in L-DOX-treated BMDMs. (**H**) GSEA plot showing enrichment of the cellular senescence pathway in L-DOX-treated BMDMs. (**I**) Heatmap showing expression levels of SASP-related factors in L-DOX-treated versus control macrophages from RNA-seq (n = 4). (**J**) Representative senescence-associated β-galactosidase staining images of control and L-DOX-treated BMDMs. Scale bars, 100 μm. (**K**) Quantification of the percentage of senescence-associated β-galactosidase-positive BMDMs corresponding to (**J**) (n = 3). (**L**) Representative Western blot showing p21 protein expression in control and L-DOX-treated BMDMs. (**M**) Densitometric quantification of p21 expression relative to β-actin corresponding to (**L**) (n = 3). Statistical test: two-tailed unpaired Student’s *t*-test (**K**,**M**). ***p* < 0.01, ****p* < 0.001, *****p* < 0.0001. Abbreviations: L-DOX, liposomal doxorubicin; RNA-seq, RNA sequencing; GO, gene ontology; GSEA, gene set enrichment analysis; NES, normalized enrichment score; SASP, senescence-associated secretory phenotype; SA-β-gal, senescence-associated β-galactosidase; BMDM, bone marrow-derived macrophage; DAPI, 4′,6-diamidino-2-phenylindole.

**Figure S4: Validation of cGAS-STING-p65-dependent NF-κB pathway.** (**A**) GSEA plot showing type I interferon pathway activation. (**B**) Densitometric quantification of p-STING relative to total STING corresponding to the Western blot shown in Fig. 4B (n = 3). (**C**) Densitometric quantification of p-TBK1 relative to total TBK1 corresponding to the Western blot shown in Fig. 4B (n = 3). (**D**) Densitometric quantification of p-IRF3 relative to total IRF3 corresponding to the Western blot shown in Fig. 4B (n = 3). (**E**) Densitometric quantification of PD-L1 relative to β-actin corresponding to the Western blot shown in Fig. 4B (n = 3). (**F**) Flow cytometry analysis of PD-L1^+^ macrophages following pharmacological inhibition at different nodes of the cGAS-STING pathway. (**G**) ChIP-qPCR analysis of p65 binding to predicted sites (p1–p3) in the PD-L1 promoter (n = 3). (**H**) Schematic of predicted NF-κB binding sites (p1–p3) and mutation design. (**I**) Representative Western blot showing p65 expression in BMDMs transfected with negative-control or p65-targeting siRNA. (**J**) Densitometric quantification of p65 expression relative to β-actin corresponding to (**I**) (n = 3). (**K**) Representative flow cytometry plot showing PD-L1 expression after NF-κB inhibition or p65 knockdown. (**L**) Representative flow cytometry histograms showing PD-L1 expression in primary hMDMs treated with L-DOX in the presence or absence of H-151 or JSH-23. (**M**) Quantification of PD-L1 mean fluorescence intensity corresponding to (**L**) (n = 3). L-DOX, H-151, and JSH-23 were used at the same concentrations as in the BMDM experiments. Statistical tests: two-tailed unpaired Student’s *t*-test (**B**–**E**); two-way ANOVA with Sidak’s correction (**G**); one-way ANOVA with Dunnett’s correction (**J**); one-way ANOVA with Sidak’s correction for indicated comparisons (**M**). **p* < 0.05, ***p* < 0.01, ****p* < 0.001, *****p* < 0.0001. ns, not significant (*p* ≥ 0.05). Abbreviations: L-DOX, liposomal doxorubicin; PD-L1, programmed death-ligand 1; cGAS, cyclic GMP-AMP synthase; STING, stimulator of interferon genes; TBK1, TANK-binding kinase 1; IRF3, interferon regulatory factor 3; NF-κB, nuclear factor kappa B; GSEA, gene set enrichment analysis; ChIP-qPCR, chromatin immunoprecipitation–quantitative PCR; BMDM, bone marrow-derived macrophage; hMDM, human monocyte-derived macrophage; TSS, transcription start site; siRNA, small interfering RNA; MFI, mean fluorescence intensity.

**Figure S5: Validating macrophage phenotype modulation by anti-PD-L1.** (**A**,**B**) t-SNE feature plots for CD14 (**A**) and CD68 (**B**) expression in GSE266919. (**C**) Sample distribution across patients in GSE266919. (**D**) Heatmap showing the average scaled expression of CD274 and representative macrophage phenotype markers across macrophage clusters from the GSE266919 dataset. Rows represent macrophage clusters, and columns represent marker genes. CD274 is shown as the PD-L1 marker but was excluded from the M2-like/immunoregulatory scoring signature. (**E**) Effect of anti-PD-L1 treatment on the expression of representative M1-like and M2-like markers in GSE116564. (**F**) Bright-field images showing morphological changes under different treatments (scale bar = 100 μm). Statistical tests: one-way ANOVA with Sidak’s correction for indicated comparisons (**E**). **p* < 0.05, ***p* < 0.01, ****p* < 0.001. Abbreviations: PD-L1, programmed death-ligand 1; L-DOX, liposomal doxorubicin; GSE, gene expression omnibus series; t-SNE, t-distributed stochastic neighbor embedding; M1, M1-like/pro-inflammatory macrophage phenotype; M2, M2-like/immunoregulatory macrophage phenotype; IgG, immunoglobulin G.

**Figure S6: CD8^+^ T Cell Subsets and Functional Validation.** (**A**) t-SNE clustering of CD8^+^ T cells from GSE266919. (**B**) Patient distribution of CD8^+^ T cells. (**C**) t-SNE clustering of CD4^+^ T cells from GSE266919. (**D**) Patient distribution of CD4^+^ T cells. (**E**) Marker gene expression for CD8^+^ T cell subsets. CD8^+^ T cells (CD3D, CD3E, CD3G, CD8A), exhausted T cells (PDCD1, LAG3, HAVCR2), proliferating T cells (MKI67, TOP2A, PCNA). (**F**) Marker gene expression for CD4^+^ T cell subsets. CD4^+^ T cells (CD3D, CD3E, CD3G, CD4), Treg cells (CD4, FOXP3, IL2RA). (**G**) Gene set score plots for GZMB and IFN-γ in CD8^+^ T cells (left) and zoomed-in view (right). Dots represent the average score per patient subgroup. (**H**) Representative flow cytometry histograms showing CD25 expression in CD8^+^ T cells under the indicated co-culture conditions. (**I**) Quantification of CD25 mean fluorescence intensity corresponding to (**H**) (n = 3). (**J**) Flow cytometry analysis showing the positivity rate (CD11c^+^) of BMDCs. (**K**) Basal cytotoxicity rate of EO771 cells cultured without CD8^+^ T cells. (**L**) Basal cytotoxicity rate of 4T1 cells cultured without CD8^+^ T cells. Statistical tests: one-way ANOVA with Sidak’s correction for indicated comparisons (**I**). ***p* < 0.01, ****p* < 0.001. ns, not significant (*p* ≥ 0.05). Abbreviations: GSE, gene expression omnibus series; t-SNE, t-distributed stochastic neighbor embedding; Treg, regulatory T cell; PD-L1, programmed death-ligand 1; L-DOX, liposomal doxorubicin; IgG, immunoglobulin G; BMDC, bone marrow-derived dendritic cell; CFSE, carboxyfluorescein succinimidyl ester; MFI, mean fluorescence intensity.

**Figure S7: *In vivo* therapeutic efficacy and immune infiltration analysis.** (**A**) Representative images of EO771 and 4T1 tumors at endpoint (n = 5). (**B**) Gating strategy in this study: single cells were selected by FSC-A/SSC-A and FSC-A/FSC-H, live cells were identified using a fixable viability dye, and immune cells were gated as CD45^+^. For T cells, CD8^+^ T cells were selected by CD3 and CD8, followed by analysis of CD69, PD-1, TIM-3, IFN-γ, and GZMB expression. For macrophages, cells were gated as F4/80^+^CD11b^+^, followed by analysis of PD-L1, CD86, and CD206 expression. Tumor cells were gated as CD45^−^EpCAM^+^. (**C**) Percentage of tumor-infiltrating macrophages in EO771 and 4T1 tumors (n = 5). (**D**) Percentage of tumor-infiltrating CD8^+^ T cells in EO771 and 4T1 tumors (n = 5). (**E**) Quantification of PD-L1 MFI in CD45^−^EpCAM^+^ tumor cells and CD45^+^CD11b^+^F4/80^+^ TAMs from control and L-DOX-treated EO771 and 4T1 tumors (n = 5). Statistical tests: one-way ANOVA with Sidak’s correction for indicated comparisons (**C**,**D**); two-way ANOVA with Sidak’s correction (**E**). **p* < 0.05, ****p* < 0.001, *****p* < 0.0001. ns, not significant (*p* ≥ 0.05). Abbreviations: L-DOX, liposomal doxorubicin; PD-L1, programmed death-ligand 1; PD-1, programmed cell death protein 1; FSC-A, forward scatter area; FSC-H, forward scatter height; SSC-A, side scatter area; FVS, fixable viability stain; TAM, tumor-associated macrophage; IgG, immunoglobulin G; MFI, mean fluorescence intensity.

**Table S1:** Public bulk transcriptomic datasets used in this study.

| **Dataset** | **Species** | **Data Type/Platform** | **Sample Size** | **Biological Source** | **Comparison/Groups** | **Paired Design** |
| --- | --- | --- | --- | --- | --- | --- |
| GSE28583 | Human | Microarray; Affymetrix U133 Plus 2.0 | 10 matched pairs | Breast tumor biopsies | Pre- vs. post-NAC | Yes |
| GSE28826 | Human | Microarray; Affymetrix U133 Plus 2.0 | 13 matched pairs | Breast tumor biopsies | Pre- vs. post-NAC | Yes |
| GSE232764 | Human | RNA-seq; Illumina NovaSeq 6000 | 8 matched pairs | Breast tumor tissue | Baseline vs. post-induction (prior to DOX/cyclophosphamide) | Yes |
| GSE260989 | Human | RNA-seq; Illumina NovaSeq 6000 | 7 matched pairs | Breast tumor tissue | Pre- vs. post-NAC | Yes |
| GSE149479 | Mouse | RNA-seq; Illumina NextSeq 500 | 12 samples | Murine mammary tumors | Control vs. DOX-treated tumors | No; independent mouse tumors |
| GSE133683 | Mouse | RNA-seq; Illumina HiSeq 2000 | 24 samples | Murine mammary tumors | Untreated vs. DOX-treated WT tumors | No; independent mouse tumors |
| GSE116564 | Mouse | RNA-seq; Illumina HiSeq 4000, paired-end 150-bp sequencing | 9 samples; n = 3 per group | Primary BMDMs from C57BL/6 mice | Medium alone, irrelevant isotype control antibody, or anti-PD-L1 antibody; 100 μg/mL for 24 h | No; independent BMDM cultures |

Note: Abbreviations: BMDM, bone marrow-derived macrophage; DOX, doxorubicin; GSE, gene expression omnibus series; NAC, neoadjuvant chemotherapy; PD-L1, programmed death ligand 1; RNA-seq, RNA sequencing; WT, wild-type; bp, base pairs.

**Table S2:** Public single-cell RNA-seq datasets used in this study.

| **Dataset** | **Species** | **Library Preparation and Sequencing Platform** | **Sample Size** | **Biological Source** | **Treatment Design** |
| --- | --- | --- | --- | --- | --- |
| GSE279219 | Human | 10x Genomics Chromium X; Chromium Next GEM Single Cell 5′ Kit v2; Illumina NovaSeq 6000 | 13 pretreatment and 11 post-treatment samples | Fresh tumor biopsies from patients with early-stage TNBC | Pretreatment vs. after one cycle of DOX plus cyclophosphamide-based NAC |
| GSE266919 | Human | 10x Genomics Chromium Next GEM Single Cell 5′ Kit; Illumina NovaSeq 6000 | 78 tumor biopsies from 44 patients | Immune cells isolated from pretreatment and/or post-treatment TNBC tumor biopsies | TNBC patients receiving various therapies, including paclitaxel, nab-paclitaxel, and their combinations with anti-PD-L1 |
| GSE191246 | Mouse | 10x Genomics Chromium, version 2 chemistry; Illumina NovaSeq 6000 | Two DOX-control pairs | FACS-sorted viable CD45^+^DAPI^−^ tumor-infiltrating immune cells from 4T1 and EMT6 tumors | Two DOX-control pairs from the 18 pooled scRNA-seq libraries (4T1/EMT6) |

Note: For GSE266919, the GEO series contains both human scRNA-seq samples and murine bulk RNA-seq experiments: Only the processed human immune-cell-specific scRNA-seq objects relevant to the present study are described here. Abbreviations: DAPI, 4′,6-diamidino-2-phenylindole; DOX, doxorubicin; FACS, fluorescence-activated cell sorting; GEO, gene expression omnibus; GSE, gene expression omnibus series; NAC, neoadjuvant chemotherapy; scRNA-seq, single-cell RNA sequencing; TNBC, triple-negative breast cancer.

**Table S3:** Canonical marker genes used for scRNA-seq cell-type annotation.

| **Dataset/Species** | **Cell Type** | **Marker Genes** |
| --- | --- | --- |
| Human/GSE279219 | Macrophages | CD68, CD14, CD163 |
|  | Dendritic cells | IRF7, IRF8, CLEC4C |
|  | Neutrophils | CSF3R, G0S2, CXCL8 |
|  | T cells | CD3D, CD3E, CD4, CD8A, CD8B |
|  | B cells | CD19, MS4A1, CD79A, CD79B |
|  | Natural killer cells | GNLY, NKG7, PRF1 |
|  | Cancer cells | EPCAM, KRT7, KRT18 |
|  | Fibroblasts | ACTA2, FGF7, MME |
|  | Endothelial cells | PTPRB, PECAM1 |
|  | Epithelial cells | KRT5, KRT14 |
|  | Plasma cells | IGHG1, MZB1, SDC1 |
|  | Adipocytes | MGLL, SAA1, SAA2 |
|  | Pericytes | PDGFRB, MCAM, RGS5 |
| Murine/GSE191246 | Monocyte-macrophages | Cd68, Cd14, Adgre1 |
|  | T cells | Cd3d, Cd3e, Cd4, Cd8a, Cd8b1 |
|  | B cells | Cd19, Ms4a1, Cd79a, Cd79b |
|  | Natural killer cells | Klrb1c, Ncr1, Nkg7 |
|  | Dendritic cells | Fcer1a, Clec9a, Clec10a |
|  | Neutrophils | Itgam, S100a8, G0s2 |

Note: Abbreviations: GSE, gene expression omnibus series; scRNA-seq, single-cell RNA sequencing.

**Table S4:** Gene sets used for macrophage phenotype scoring.

| **Gene Set** | **Marker Genes** |
| --- | --- |
| M1_Polarization | IL12, IL23, TNF, IL6, CD86, IL1B, MARCO, NOS2, CD64, CD80, CXCL9, CXCL10, CXCL11, IL1A, CCL5, IRF5, IRF1, CD40, IDO1, KYNU, CCR7 |
| M2_Polarization | ARG1, ARG2, IL10, CD32, CD163, CD23, CD200R1, PDCD1LG2, MARCO, CSF1R, CD206, IL1RN, IL1R2, IL4R, CCL4, CCL13, CCL20, CCL17, CCL18, CCL22, CCL24, LYVE1, VEGFA, VEGFB, VEGFC, VEGFD, EGF, CTSA, CTSB, CTSC, CTSD, TGFB1, TGFB2, TGFB3, MMP14, MMP19, MMP9, CLEC7A, WNT7B, FASL, TNFSF12, TNFSF8, CD276, VTCN1, MSR1, FN1, IRF4 |
| Anti_inflammatory | IL1RN, IL10, IL4, IL11, IL13, TGFB1, TNFRSF1A, TNFRSF1B, IL1R2, IL18BP |
| Pro_inflammatory | IL1B, TNF, CCL2, CCL3, CCL5, CCL7, CCL8, CCL13, CCL17, CCL22 |

Note: Gene sets were compiled based on established macrophage polarization and inflammatory signatures reported in previous studies [43,44]. Abbreviations: M1, M1-like/pro-inflammatory macrophage phenotype; M2, M2-like/immunoregulatory macrophage phenotype.

**Table S5:** Chemotherapeutic agents and inhibitors.

| **Reagent** | **Source** | **Cat No./Approval No.** | **Usage** |
| --- | --- | --- | --- |
| Pifithrin-α | Beyotime Biotechnology, Shanghai, China | S1816-5 mg | 20 μM |
| KU-55933 | Beyotime Biotechnology, Shanghai, China | SC9136-10 mM | 20 μM |
| H-151 | MedChemExpress, Monmouth Junction, NJ, USA | HY-112693 | 500 nM |
| GSK8612 | MedChemExpress, Monmouth Junction, NJ, USA | HY-111941 | 500 nM |
| G140 | MedChemExpress, Monmouth Junction, NJ, USA | HY-133916 | 2 μM |
| L-DOX | Changzhou Jinyuan Pharmaceutical Manufacturing Co., Ltd., Changzhou, Jiangsu, China | NMPA approval No. H20123273 | 1 μM for *in vitro* macrophage experiments; 5 mg/kg weekly for *in vivo* treatment |
| DOX | Shanxi Pude Pharmaceutical Co., Ltd., Datong, Shanxi, China | NMPA approval No. H14023879 | 1 μM |
| PTX | Pfizer Australia Pty Ltd., Sydney, NSW, Australia; manufactured by Hospira Australia Pty Ltd., Mulgrave, VIC, Australia | NMPA approval Nos. HJ20150350/HJ20150351 | 1 μM |
| DTX | Jiangsu Hengrui Pharmaceuticals Co., Ltd., Lianyungang, Jiangsu, China | NMPA approval No. H20020543 | 1 μM |
| Nab-PTX | CSPC Ouyi Pharmaceutical Co., Ltd., Shijiazhuang, Hebei, China | NMPA approval No. H20183044 | 1 μM |
| L-PTX | Nanjing Luye Pharmaceutical Co., Ltd., Nanjing, Jiangsu, China | NMPA approval No. H20030357 | 1 μM |

Note: Abbreviations: DOX, doxorubicin; DTX, docetaxel; L-DOX, liposomal doxorubicin; L-PTX, liposomal paclitaxel; Nab-PTX, nanoparticle albumin-bound paclitaxel; NMPA, national medical products administration; PTX, paclitaxel.

**Table S6:** Flow cytometry antibodies and reagents.

| **Antibodies** | **Source (City, Country)** | **Cat No.** | **Clone** | **Dilution/Concentration** |
| --- | --- | --- | --- | --- |
| PD-L1 | Elabscience, Wuhan, Hubei, China | E-AB-F1132C | 10F.9G2 | 1:20 |
| CD16/32 | BioLegend, San Diego, CA, USA | 101301 | 93 | 1:100 |
| CD3 | Elabscience, Wuhan, Hubei, China | E-AB-F1013D | 17A2 | 1:20 |
| CD8a | BD Biosciences, Franklin Lakes, NJ, USA | 562283 | 53-6.7 | 1:100 |
| CD25 | Proteintech Group, Wuhan, Hubei, China | FITC-65137 | PC61.5 | 1:100 |
| CD69 | Proteintech Group, Wuhan, Hubei, China | PE-65105 | H1.2F3 | 1:100 |
| IFN-γ | Elabscience, Wuhan, Hubei, China | E-AB-F1101C | XMG1.2 | 1:20 |
| GZMB | BioLegend, San Diego, CA, USA | 372212 | QA16A02 | 1:20 |
| CFSE | Elabscience, Wuhan, Hubei, China | E-CK-A345 | / | 5 μM |
| PD-1 | Proteintech Group, Wuhan, Hubei, China | FITC-65142 | RMP1-30 | 1:100 |
| TIM3 | Elabscience, Wuhan, Hubei, China | E-AB-F1192UE | RMT3-23 | 1:100 |
| Fixable Viability Dye | BioLegend, San Diego, CA, USA | 423101 | / | 1:500 |
| CD11b | BD Biosciences, Franklin Lakes, NJ, USA | 560456 | M1/70 | 1:50 |
| F4/80 | Elabscience, Wuhan, Hubei, China | E-AB-F0995D | CI:A3-1 | 1:20 |
| CD86 | eBioscience, Thermo Fisher Scientific, Waltham, MA, USA | 15-0862-82 | GL1 | 1:100 |
| CD206 | BioLegend, San Diego, CA, USA | 141707 | C068C2 | 1:100 |
| CD45 | Elabscience, Wuhan, Hubei, China | E-AB-F1136N | 30-F11 | 1:20 |
| CD326/EpCAM | BioLegend, San Diego, CA, USA | 118227 | G8.8 | 1:100 |
| Annexin V-FITC/PI | Vazyme Biotech, Nanjing, Jiangsu, China | A211-02 | / | 1:20 |
| Human CD45 | BioLegend, San Diego, CA, USA | 304036 | HI30 | 1:20 |
| Human CD64 | BioLegend, San Diego, CA, USA | 305020 | 10.1 | 1:20 |
| Human CD11b | BioLegend, San Diego, CA, USA | 301310 | ICRF44 | 1:20 |
| Human PD-L1/CD274 | BioLegend, San Diego, CA, USA | 329718 | 29E.2A3 | 1:20 |

Note: CFSE was used at a final working concentration of 5 μM rather than at a dilution ratio. Abbreviations: CFSE, carboxyfluorescein succinimidyl ester; FITC, fluorescein isothiocyanate; PD-1, programmed cell death protein 1; PD-L1, programmed death-ligand 1; PE, phycoerythrin; PI, propidium iodide.

**Table S7:** Western blot antibodies.

| **Antibodies** | **Source (City, Country)** | **Cat No.** | **Usage** |
| --- | --- | --- | --- |
| β-actin | Proteintech Group, Wuhan, Hubei, China | 66009-1-Ig | 1:2000 |
| PD-L1 | Proteintech Group, Wuhan, Hubei, China | 66248-1-Ig | 1:2000 |
| ATM | Boster Biological Technology, Pleasanton, CA, USA | BA0655-2 | 1:2000 |
| p-ATM | Boster Biological Technology, Pleasanton, CA, USA | BM4008 | 1:2000 |
| p53 | ABclonal Technology, Wuhan, Hubei, China | A19585 | 1:30000 |
| γ-H2AX | Cell Signaling Technology, Danvers, MA, USA | 80312S | 1:2000 |
| p21 | Proteintech Group, Wuhan, Hubei, China | 28248-1-AP | 1:2000 |
| STING | HUABIO, Hangzhou, Zhejiang, China | HA722832 | 1:5000 |
| p-STING | Cell Signaling Technology, Danvers, MA, USA | 50907S | 1:1000 |
| TBK1 | ABclonal Technology, Wuhan, Hubei, China | A3458 | 1:1000 |
| p-TBK1 | Abcam, Cambridge, UK | AB109272 | 1:3000 |
| IRF3 | HUABIO, Hangzhou, Zhejiang, China | ET1612-14 | 1:1000 |
| p-IRF3 | HUABIO, Hangzhou, Zhejiang, China | HA722772 | 1:1000 |
| p65 | Proteintech Group, Wuhan, Hubei, China | 10745-1-AP | 1:1000 |
| p-p65 | Proteintech Group, Wuhan, Hubei, China | 82335-1-RR | 1:3000 |

Note: Prestained Protein Ladder (Thermo Fisher Scientific, 26616, Waltham, MA, USA). Abbreviations: ATM, ataxia-telangiectasia mutated; IRF3, interferon regulatory factor 3; PD-L1, programmed death-ligand 1; STING, stimulator of interferon genes; TBK1, TANK-binding kinase 1.

**Table S8:** ChIP-qPCR primers and NF-κB-binding sites in the mouse *Cd274* promoter.

| **Site** | **Motif Position Relative to TSS** | **Genomic Coordinates** | **WT Motif** | **Amplicon Region Relative to TSS** | **Forward Primer (5′–3′)** | **Reverse Primer (5′–3′)** |
| --- | --- | --- | --- | --- | --- | --- |
| p1 | −1980 to −1971 bp | NC_000085.7:29337448–29337457 | AGGAAATCCC | 1984 to −1860 bp | GTAGAGACAGGGTCTCAC | CCTGGCCAATTTTTAAATTT |
| p2 | −1164 to −1155 bp | NC_000085.7:29338264–29338273 | GGGAAATCTC | −1174 to −1045 bp | TCTTTTCCCATATTTTCCAGG | GACACAGTTGAGACCATGTCTC |
| p3 | −475 to −466 bp | NC_000085.7:29338953–29338962 | GGTATTTCCC | −485 to −355 bp | TAGCCCTGGCTGTCCTGGAAC | GCCACACACCCAGCCAATTTT |

Note: Abbreviations: ChIP-qPCR, chromatin immunoprecipitation–quantitative PCR; NF-κB, nuclear factor kappa B; TSS, transcription start site; WT, wild-type.

**Table S9:** siRNA sequences.

|  | **SS Sequence (5′-3′)** | **AS Sequence (5′-3′)** |
| --- | --- | --- |
| siRNA1 | GGAGUACCCUGAAGCUAUAAC | UAUAGCUUCAGGGUACUCCAU |
| siRNA2 | GGCGAGAGAAGCACAGAUACC | UAUCUGUGCUUCUCUCGCCAG |

Note: Abbreviations: AS, antisense strand; SS, sense strand.
